# A Polynesian-specific copy number variant encompassing the MICA gene associates with gout

**DOI:** 10.1093/hmg/ddac094

**Published:** 2022-04-22

**Authors:** Ke Wang, Murray Cadzow, Matt Bixley, Megan P Leask, Marilyn E Merriman, Qiangzhen Yang, Zhiqiang Li, Riku Takei, Amanda Phipps-Green, Tanya J Major, Ruth Topless, Nicola Dalbeth, Frances King, Rinki Murphy, Lisa K Stamp, Janak de Zoysa, Zhuo Wang, Yongyong Shi, Tony R Merriman

**Affiliations:** Bio-X Institutes, Key Laboratory for the Genetics of Developmental and Neuropsychiatric Disorders (Ministry of Education), Shanghai Jiao Tong University, Shanghai 200030, People’s Republic of China; Department of Biochemistry, University of Otago, Dunedin 9054, New Zealand; Department of Biochemistry, University of Otago, Dunedin 9054, New Zealand; Department of Biochemistry, University of Otago, Dunedin 9054, New Zealand; Division of Clinical Immunology and Rheumatology, University of Alabama at Birmingham, Birmingham, AL 35233, USA; Department of Biochemistry, University of Otago, Dunedin 9054, New Zealand; Bio-X Institutes, Key Laboratory for the Genetics of Developmental and Neuropsychiatric Disorders (Ministry of Education), Shanghai Jiao Tong University, Shanghai 200030, People’s Republic of China; Bio-X Institutes, Key Laboratory for the Genetics of Developmental and Neuropsychiatric Disorders (Ministry of Education), Shanghai Jiao Tong University, Shanghai 200030, People’s Republic of China; Biomedical Sciences Institute of Qingdao University (Qingdao Branch of SJTU Bio-X Institutes), Qingdao University, Qingdao 266003, China; Department of Biochemistry, University of Otago, Dunedin 9054, New Zealand; Division of Clinical Immunology and Rheumatology, University of Alabama at Birmingham, Birmingham, AL 35233, USA; Department of Biochemistry, University of Otago, Dunedin 9054, New Zealand; Department of Biochemistry, University of Otago, Dunedin 9054, New Zealand; Department of Biochemistry, University of Otago, Dunedin 9054, New Zealand; Department of Medicine, University of Auckland, Auckland 1023, New Zealand; Ngati Porou Hauora Charitable Trust, Te Puia Springs, New Zealand; Department of Medicine, University of Auckland, Auckland 1023, New Zealand; Department of Medicine, University of Otago, Christchurch 8013, New Zealand; Department of Medicine, University of Auckland, Auckland 1023, New Zealand; Bio-X Institutes, Key Laboratory for the Genetics of Developmental and Neuropsychiatric Disorders (Ministry of Education), Shanghai Jiao Tong University, Shanghai 200030, People’s Republic of China; Bio-X Institutes, Key Laboratory for the Genetics of Developmental and Neuropsychiatric Disorders (Ministry of Education), Shanghai Jiao Tong University, Shanghai 200030, People’s Republic of China; Biomedical Sciences Institute of Qingdao University (Qingdao Branch of SJTU Bio-X Institutes), Qingdao University, Qingdao 266003, China; Department of Biochemistry, University of Otago, Dunedin 9054, New Zealand; Division of Clinical Immunology and Rheumatology, University of Alabama at Birmingham, Birmingham, AL 35233, USA

## Abstract

Gout is of particularly high prevalence in the Māori and Pacific (Polynesian) populations of Aotearoa New Zealand (NZ). Here, we investigated the contribution of common population-specific copy number variation (CNV) to gout in the Aotearoa NZ Polynesian population. Microarray-generated genome-wide genotype data from Aotearoa NZ Polynesian individuals with (*n* = 1196) and without (*n* = 1249) gout were analyzed. Comparator population groups were 552 individuals of European ancestry and 1962 of Han Chinese ancestry. Levels of circulating major histocompatibility complex (MHC) class I polypeptide-related sequence A (MICA) were measured by enzyme-linked immunosorbent assay. Fifty-four CNV regions (CNVRs) appearing in at least 10 individuals were detected, of which seven common (>2%) CNVRs were specific to or amplified in Polynesian people. A burden test of these seven revealed associations of insertion/deletion with gout (odds ratio (OR) 95% confidence interval [CI] = 1.80 [1.01; 3.22], *P* = 0.046). Individually testing of the seven CNVRs for association with gout revealed nominal association of CNVR1 with gout in Western Polynesian (Chr6: 31.36–31.45 Mb, OR = 1.72 [1.03; 2.92], *P* = 0.04), CNVR6 in the meta-analyzed Polynesian sample sets (Chr1: 196.75–196.92 Mb, OR = 1.86 [1.16; 3.00], *P* = 0.01) and CNVR9 in Western Polynesian (Chr1: 189.35–189.54 Mb, OR = 2.75 [1.15; 7.13], *P* = 0.03). Analysis of European gout genetic association data demonstrated a signal of association at the CNVR1 locus that was an expression quantitative trait locus for *MICA*. The most common CNVR (CNVR1) includes deletion of the *MICA* gene, encoding an immunomodulatory protein. Expression of MICA was reduced in the serum of individuals with the deletion. In summary, we provide evidence for the association of CNVR1 containing *MICA* with gout in Polynesian people, implicating class I MHC-mediated antigen presentation in gout.

## Introduction

Gout is a common complex metabolic and inflammatory disease caused by hyperuricemia and immune response to the deposition of monosodium urate (MSU) crystals in and around body tissues, particularly joints (1). The clinical features consist of intermittent gout flares, chronic gouty arthritis, tophi and increased prevalence of comorbidities that include hypertension, type 2 diabetes mellitus, chronic kidney disease, dyslipidemia and cardiovascular disease (1–5). The pathogenesis of gout requires progression through several phases: from hyperuricemia to the deposition of MSU crystals to subsequent immune response to these crystals (6). In some individuals, the crystals cause a toll-like receptor-mediated formation and activation of the NLRP3 inflammasome in monocytes and production of interleukin-1β that drives the gout flare.

Genome-wide association studies (GWAS) that focus on single-nucleotide variants (SNVs) have provided considerable insight into the molecular pathogenesis of hyperuricemia (7–9), although GWAS in gout have been more limited (10). However, common SNVs explain only a relatively small fraction of the heritability of gout, with genome-wide heritability estimates of around 0.3 (11). Structural variants are defined as genomic rearrangements affecting >50 bp of sequence, and they impact a greater proportion of the genome than SNVs. Therefore, they have a greater likelihood of having an impact on molecular function and phenotype. Structural variants are firmly implicated in common disease including schizophrenia (12), Alzheimer’s disease (13), intellectual disabilities (14), autism (15,16), Crohn’s disease (17,18) and rheumatic diseases (19–21). In gout and hyperuricemia, single small studies have reported that copy number variations (CNVs) of *ABCF1, IL17REL* and *FCGR3A* are associated with the risk of gout in the Chinese population (22), and CNV upstream of *SLC2A9* associates with urate levels in Europeans (23), suggesting a role for genomic structural variation in the pathogenesis of gout.

The prevalence of gout among Māori (the indigenous people of Aotearoa New Zealand) and New Zealand Pacific adults is 8% and 14%, respectively, compared to 4% for non-Māori and non-Pacific people (24). While structural inequities contribute to increased prevalence and poorer outcomes (25), research on population-specific genetic variants associated with gout is important to provide insights into the disparities between different populations, especially in populations currently under-served with respect to participation in genetic studies. It is becoming clear that genetic variation that is specific to Pacific populations does exist and that such variation contributes to the pathogenesis of gout (26–28) and other metabolic diseases (29). Our primary hypothesis was that there are Polynesian-specific (or amplified) CNV regions (CNVRs) associated with gout and which may contribute to the increased prevalence of gout in Polynesian populations. Therefore, we aimed to identify Polynesian-specific CNVs contributing to gout using genome-wide genotype data in a total of 1196 people with and 1249 people without gout.

## Results

### Discovery and characteristics of the CNVs

**Figure 1:**
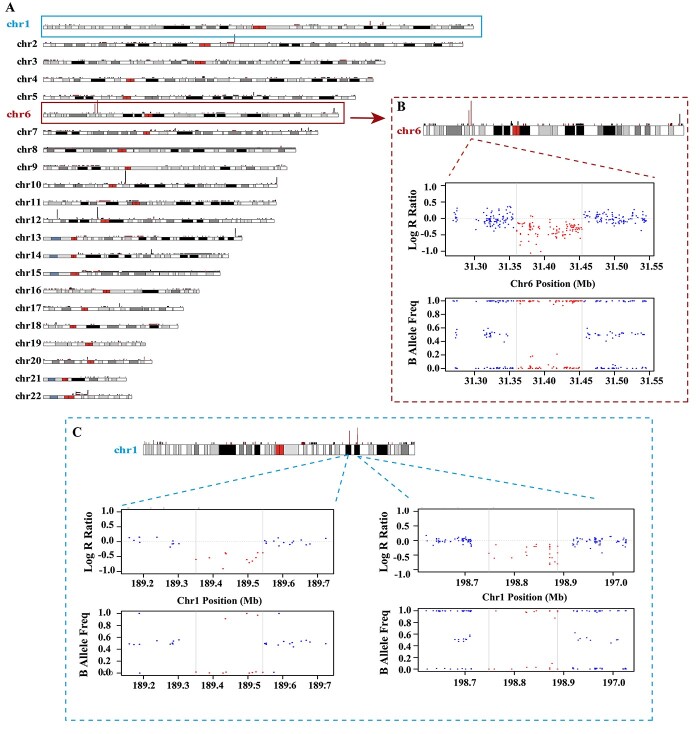
(**A**) Genomic distribution of CNVs. Red lines show copy number loss and black lines show copy number gain. (**B**, **C**) Example of deletions in CNV regions.

**Figure 2:**
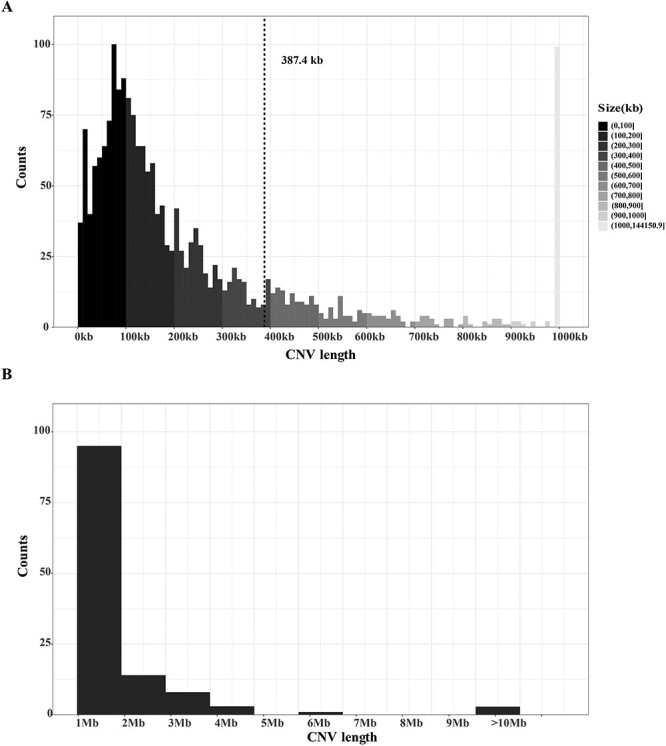
The size distribution of CNVs. (**A**) Size (in kb) compared to the number of CNVs. The CNVs were grouped into 10 kb windows. For the purposes of visualization, large CNVs (>1 Mb) were classified to 1 Mb. The dotted line represents the mean CNV size. (**B**) Distribution of large CNVs (>1 Mb).

Aotearoa NZ Polynesian and European participants that had CNV count ≤312 (85th percentile) were used for CNV identification and analysis. Among 4353 CNV calls detected after the quality control filtering, 2607 (1427 duplications and 1180 deletions) were found in people with gout and 1746 (899 duplications and 847 deletions) in people without gout. The distribution of CNVs along each of the chromosomes is shown in Figure 1A. The identified CNVs ranged in size from 5007 to 144 150 872 bp (144 Mb), with an average length of 385.3 kb (Fig. 2). There were three CNVs >10 Mb that were present in two European individuals (Supplementary Material, Fig. S1)—duplications at Chr8: 2.09–146.25 Mb, Chr15: 31.53–43.38 Mb and Chr15: 45.57–102.40 Mb (the same individual had both Chr15 duplications), representing apparent trisomy of chromosome 8 and chromosome 15, respectively. We did not further investigate these individuals as they did not present with significant health issues and the observed data may be a result of artefact and/or low-level somatic mosaicism. Supplementary Material, Table S1 lists all 54 CNVRs that were present in ≥10 individuals, including details of each copy number type that PennCNV reported. Representative examples of LRR and BAF plots are shown in Supplementary Material, Figure S2 for the 20 most common CNVRs. Comparing length of all types of CNVs between gout and non-gout revealed no significant differences with respect to the average counts and length of CNV (Supplementary Material, Fig. S3).

### Testing Polynesian-specific and -amplified CNVR for association with gout

Specifically, we examined the seven CNVRs of >2% prevalence in Polynesian that were either specific to Polynesian [CNVR1 (Chr6: 31.36–31.45 Mb), CNVR2 (Chr6: 29.92–29.94 Mb), CNVR6 (Chr1: 196.75–196.92 Mb), CNVR8 (Chr13: 111.17–111.33 Mb), CNVR9 (Chr1: 189.35–189.54 Mb), CNVR15 (Chr14: 62.63–62.77 Mb)] or were amplified in Polynesian [<1% in European and Chinese; CNVR7 (Chr2: 110.86–110.98 Mb)]. A test for association with gout was performed by comparing individuals with a CNV in any of the seven reported regions (pooled) to those who were diploid for all seven CNVRs. These seven CNVRs were associated with gout in Polynesian using Model 2 [odds ratio (OR) = 1.80, *P* = 0.046] (Table 1), with the strongest association in the Western Polynesian sample set (OR = 2.43, *P* = 2.1E−06).

**Table 1:** Association with gout of the burden of seven common specific or amplified CNVRs in the Aotearoa NZ Polynesian sample sets

Ethnicity	Gout		Control		Unadjusted		Adjusted (Model 1)		Adjusted (Model 2)	
	With CNV	Without CNV	With CNV	Without CNV	OR (95% CI)	*P*	OR (95% CI)	*P*	OR (95% CI)	*P*
EP	255 (38.2%)	413 (61.8%)	221 (34.7%)	416 (65.3%)	1.39 (1.12–1.73)	**0.003**	1.3 (1.01–1.67)	**0.039**	1.21 (0.94–1.58)	0.14
WP	224 (57.3%)	167 (42.7%)	123 (46.1%)	144 (53.9%)	2.44 (1.83–3.25)	**1.3E-09**	2.77 (1.96–3.93)	**1.0E-08**	2.43 (1.69–3.52)	**2.1E-06**
EPWP	23 (56.1%)	18 (43.9%)	22 (40%)	33 (60%)	2.34 (1.08–5.16)	**0.032**	3.18 (1.33–8.06)	**0.011**	2.48 (0.79–8.13)	0.1231
OP-meta	502 (45.6%)	598 (54.4%)	366 (38.2%)	593 (61.8%)	1.91 (2.22–3.01)	**0.005** *P*_het_ < 0.01, *I*^2^ = 80%	2.12 (1.13–3.95)	**0.019** *P*_het_ < 0.01, *I*^2^ = 85%	1.80 (1.01–3.22)	**0.046** *P*_het_ < 0.01, *I*^2^ = 80%

EP, Eastern Polynesian; EPWP, Mixed Eastern–Western Polynesian; WP, Western Polynesian; OP-meta, WP, EP and EPWP were combined by meta-analysis with random effects; CI, confidence intervals; OR, odds ratio; Model 1, adjusted by age and sex; Model 2, Model 1 plus adjustment by batches and PCs 1–10. *P*-values < 0.05 are shown in bold.

The seven CNVRs were then individually tested for association with gout in the Polynesian sample set. Three provided evidence for association with gout at a nominal level of significance in the fully adjusted model: CNVR1 (OR = 1.72, *P* = 0.04, Western Polynesian), CNVR6 (OR = 1.86, *P* = 0.01, meta-analyzed Polynesian) and CNVR9 (OR = 2.75, *P* = 0.03, Western Polynesian) (Table 2). Linear regression testing for association with serum urate level in controls provided no evidence for association of any of the three CNVRs, even in the unadjusted models (Supplementary Material, Table S2). Association with gout but not serum urate level is consistent with a possible role of the deletions in the progression from hyperuricemia to gout.

**Table 2:** Logistic regression association analysis with gout

CNVR	Ethnicity	Cases			Controls			Unadjusted		Adjusted (Model 1)		Adjusted (Model 2)			Gene list
		CN = 0	CN = 1	CN = 3	CN = 0	CN = 1	CN = 3	OR (95% CI)	*P*	OR (95% CI)	*P*	OR (95% CI)	*P*		
CNVR1	WP	3 (0.8%)	60 (15.4%)	1 (0.3%)	1 (0.4%)	38 (14.2%)	0 (0%)	1.60 (1.05–2.45)	**0.03**	1.84 (1.12–3.07)	**0.02**	1.72 (1.03–2.92)	**0.04**	*P*_het_ = 0.13, *I*^2^ = 52% *P*_het*1*_ = 0.05, *I*^2^ = 68% *P*_het2_ = 0.07, *I*^2^ = 62%	HCG26,HCP5,MICA
	EP	11 (1.7%)	104 (15.6%)	6 (0.9%)	4 (0.6%)	88 (13.8%)	9 (1.4%)	1.36 (1.02–1.81)	**0.04**	1.16 (0.84–1.61)	0.37	1.10 (0.79–1.54)	0.57		
	EPWP	0 (0%)	9 (22.0%)	0 (0%)	0 (0%)	3 (5.5%)	0 (0%)	5.66 (1.57–26.74)	**0.01**	6.06 (1.53–31.69)	**0.02**	6.33 (1.24–43.16)	**0.04**		
	OP-meta	14 (1.3%)	173 (15.7%)	7 (0.6%)	5 (0.5%)	129 (13.5%)	9 (0.9%)	1.63 (1.07–2.48)	**0.02**	1.73 (0.94–3.17)	0.08	1.56 (0.87–2.78)	0.14		
CNVR2	WP	12 (3.1%)	62 (15.9%)	0 (0%)	8 (3.0%)	40 (15.0%)	0 (0%)	1.50 (1.02–2.23)	**0.04**	1.45 (0.91–2.34)	0.12	1.24 (0.76–2.03)	0.40	*P*_het_ = 0.20, *I*^2^ = 38% *P*_het1_ = 0.47, *I*^2^ = 0% *P*_het2_ = 0.44, *I*^2^ = 0%	HLA-G,HLA-H,HLA-J
	EP	3 (0.5%)	56 (8.4%)	6 (0.9%)	3 (0.5%)	65 (10.2%)	6 (0.9%)	0.95 (0.67–1.35)	0.77	0.98 (0.65–1.48)	0.93	0.91 (0.6–1.38)	0.64		
	EPWP	0 (0%)	6 (14.6%)	0 (0%)	2 (3.6%)	9 (16.4%)	0 (0%)	0.83 (0.27–2.38)	0.74	1.1 (0.31–3.62)	0.88	0.52 (0.11–2.25)	0.39		
	OP-meta	15 (1.4%)	124 (11.3%)	6 (0.6%)	13 (1.4%)	114 (11.9%)	6 (0.6%)	1.14 (0.88–1.47)	0.31	1.16 (0.86–1.56)	0.34	1.01 (0.74–1.37)	0.96		
CNVR6	WP	18 (4.6%)	24 (6.1%)	0 (0%)	11 (4.1%)	12 (4.5%)	0 (0%)	1.75 (1.04–3.01)	**0.04**	1.71 (0.93–3.23)	0.09	1.71 (0.91–3.31)	0.11	*P*_het_ = 0.45, *I*^2^ = 0% *P*_het1_ = 0.50, *I*^2^ = 0% *P*_het2_ = 0.83, *I*^2^ = 0%	CFHR1,CFHR2,CFHR3,CFHR4
	EP	13 (2.0%)	12 (1.8%)	0 (0%)	4 (0.6%)	11 (1.7%)	0 (0%)	1.84 (0.97–3.6)	0.07	1.92 (0.93–4.1)	0.08	1.92 (0.91–4.15)	0.09		
	EPWP	3 (7.3%)	3 (7.3%)	0 (0%)	0 (0%)	2 (3.6%)	0 (0%)	5.29 (1.15–37.34)	**0.05**	5.34 (1.02–41.76)	0.07	3.21 (0.51–27.77)	0.23		
	OP-meta	34 (3.1%)	39 (3.6%)	0 (0%)	15 (1.6%)	25 (2.6%)	0 (0%)	1.90 (1.28–2.83)	**2.0E-03**	1.93 (1.22–3.05)	**5.0E-03**	1.86 (1.16–3.00)	**0.01**		
CNVR7	WP	0 (0%)	0 (0%)	44 (11.3%)	0 (0%)	0 (0%)	25 (9.4%)	1.69 (1.02–2.85)	**0.05**	2.19 (1.19–4.14)	**0.01**	1.76 (0.95–3.37)	0.08	*P*_het_ = 0.67, *I*^2^ = 0% *P*_het1_ = 0.62, *I*^2^ = 0% *P*_het2_ = 0.80, *I*^2^ = 0%	LINC00116,MALL,NPHP1
	EP	0 (0%)	3 (0.4%)	12 (1.8%)	0 (0%)	1 (0.2%)	8 (1.3%)	1.83 (0.81–4.38)	0.16	1.45 (0.58–3.92)	0.44	1.20 (0.47–3.31)	0.71		
	EPWP	0 (0%)	0 (0%)	3 (7.3%)	0 (0%)	0 (0%)	1 (1.8%)	4.98 (0.61–102.41)	0.17	4.67 (0.47–112.51)	0.23	2.00 (0.17–53.25)	0.61		
	OP-meta	0 (0%)	3 (0.3%)	59 (5.4%)	0 (0%)	1 (0.1%)	34 (3.5%)	1.79 (1.17–2.75)	**8.0E-03**	2.01 (1.21–3.33)	**7.0E-03**	1.58 (0.94–2.66)	0.083		
CNVR8	WP	0 (0%)	0 (0%)	35 (9.0%)	0 (0%)	0 (0%)	19 (7.1%)	1.75 (1–3.17)	0.06	2.27 (1.16–4.57)	**0.02**	1.84 (0.94–3.71)	0.08	*P*_het_ = 0.29, *I*^2^ = 18% *P*_het1_ = 0.18, *I*^2^ = 42% *P*_het2_ = 0.18, *I*^2^ = 42%	CARKD,CARS2,RAB20
	EP	0 (0%)	2 (0.3%)	17 (2.5%)	0 (0%)	0 (0%)	19 (3.0%)	1.09 (0.57–2.08)	0.80	0.98 (0.46–2.1)	0.97	0.86 (0.4–1.89)	0.71		
	EPWP	0 (0%)	0 (0%)	1 (2.4%)	0 (0%)	0 (0%)	5 (9.1%)	0.38 (0.02–2.66)	0.39	0.52 (0.03–4.22)	0.58	0.24 (0.01–3.1)	0.32		
	OP-meta	0 (0%)	2 (0.2%)	53 (4.8%)	0 (0%)	0 (0%)	43 (4.5%)	1.35 (0.89–2.06)	0.16	1.48 (0.90–2.42)	0.12	1.25 (0.75–2.07)	0.39		
CNVR9	WP	0 (0%)	28 (7.2%)	0 (0%)	1 (0.4%)	9 (3.4%)	0 (0%)	1.28 (0.73–2.24)	**9.0E-03**	3.00 (1.29–7.55)	**0.01**	2.75 (1.15–7.13)	**0.03**	*P*_het_ = 0.25, *I*^2^ = 27% *P*_het1_ = 0.22, *I*^2^ = 34% *P*_het2_ = 0.37, *I*^2^ = 0%	None
	EP	2 (0.3%)	25 (3.7%)	1 (0.1%)	0 (0%)	24 (3.8%)	0 (0%)	2.68 (1.33–5.86)	0.39	1.25 (0.67–2.36)	0.48	1.24 (0.65–2.37)	0.51		
	EPWP	0 (0%)	2 (4.9%)	0 (0%)	0 (0%)	1 (1.8%)	0 (0%)	3.24 (0.3–70.95)	0.34	4.40 (0.36–106.27)	0.26	2.08 (0.09–84.61)	0.67		
	OP-meta	2 (0.2%)	55 (5%)	1 (0.1%)	0 (0%)	34 (3.5%)	0 (0%)	1.71 (1.11–2.64)	**0.02**	1.74 (1.06–2.88)	**0.03**	1.63 (0.97–2.73)	0.07		
CNVR15	WP	0 (0%)	0 (0%)	9 (2.3%)	0 (0%)	0 (0%)	6 (2.3%)	1.38 (0.49–4.16)	0.54	0.84 (0.27–2.8)	0.77	0.78 (0.24–2.7)	0.69	*P*_het_ = 0.60, *I*^2^ = 0% *P*_het1_ = 0.18, *I*^2^ = 44% *P*_het2_ = 0.17, *I*^2^ = 46%	None
	EP	0 (0%)	0 (0%)	21 (3.1%)	0 (0%)	0 (0%)	12 (1.9%)	1.93 (0.96–4.07)	0.07	2.19 (0.98–5.1)	0.06	2.15 (0.95–5.09)	0.07		
	EPWP	0 (0%)	0 (0%)	0 (0%)	0 (0%)	0 (0%)	3 (5.5%)	N.A.	-	N.A.	-	N.A.	-		
	OP-meta	0 (0%)	0 (0%)	30 (2.7%)	0 (0%)	0 (0%)	21 (2.2%)	1.73 (0.96–3.13)	0.07	1.59 (0.82–3.09)	0.18	1.54 (0.78–3.05)	0.22		

*P*-values < 0.05 are shown in bold; CN, copy number (only reported genotype were shown). EP, Eastern Polynesian; EPWP Mixed Eastern–Western Polynesian; WP, Western Polynesian; OP-meta, WP, EP and EPWP were combined by meta-analysis with random effects for CNVR1 and fixed effects for the other CNVRs; CI, confidence intervals; OR, odds ratio; N.A., not available; %, the frequency of case/control; Model 1, adjusted by age and sex; Model 2, Model 1 plus adjustment by batches and PCs 1–10; *P*_het_, *P*_het1*,*_  *P*_het2*,*_ heterogeneity of *P* for unadjusted, Model 1 and Model 2, respectively, in the meta-analysis.

### Further validation of CNVR1, CNVR6 and CNVR9

Examination of the CNVR1, CNVR6 and CNVR9 genomic regions in the Database of Genomic Variants (DGV) revealed that these regions are established CNV sites with multiple reported losses and gains (Supplementary Material, Table S3). There are 6, 9 and 37 DGV Gold Standard Variants that overlap with CNVR1, CNVR9 and CNVR6, respectively (Supplementary Material, Table S3).

A total of 91 Polynesian participants with both microarray and 30X whole genome sequencing (WGS) data were used to validate CNVR1, CNVR6 and CNVR9. Read depth of these individuals was plotted from WGS data. The average consistency of the copy number calls in microarray and WGS data was 94% (Supplementary Material, Table S4, 96%, 88% and 97%, respectively), indicating that our CNV calls were reliable. Representative examples of WGS read-depth plots are shown in Supplementary Material, Figure S4.

### Association analysis of loci encompassing CNVR1, CNVR6 and CNVR9 with gout in the UK Biobank

We used gout GWAS SNV data in a European dataset to test the CNVR1, CNVR6 and CNVR9 loci for association with gout (UK Biobank; 7131 cases and 325 239 controls). Supplementary Material, Figure S5 presents locus zoom plots for each locus. There was a signal of association in the CNVR1 region, but not CNVR6 or CNVR9. (The lead SNV at CNVR6 with *P* < 1 × 10^–4^ (rs143765601) was uncommon with no gout-associated SNVs in linkage disequilibrium and therefore was considered unreliable.) At CNVR1, there appeared to be two signals of genetic association—one signal was marked by top associated variant *rs3016018* and the second marked by *rs9265955.* We tested each for association with expression of genes within CNVR1 using the Gene and Tissue Expression (GTEx) resource (Supplementary Material, Fig. S6). *Rs3016018* is associated with the expression of multiple protein-coding genes outside CNVR1 (e.g. *HLA-S, PSORS1C3, NOTCHC4, C4B*); however, *rs9265955*, which also associated with the expression of multiple genes outside of CNVR1, is also associated with the expression of *MHC class I polypeptide-related sequence A* (*MICA*), the single protein-coding gene within CNVR1 (*P* = 3.6 × 10^−5^). The gout risk (C) allele of *rs9265955* increased the expression of *MICA.* This evidence from the UK Biobank supports the *MICA* locus as involved in the etiology of gout.

### Physical connectedness within CNVR1

**Figure 3:**
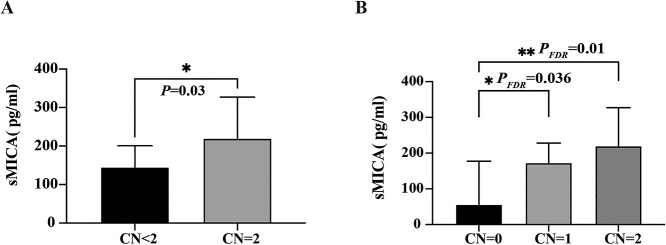
Genomic connectedness at CNVR1. Schematic of UCSC genome tracks at the CNVR1 deletion encompassing the *MICA::HCP5* locus. (**top**) Hi-C genome track for GM12878 cells at CNVR1. Hi-C fragments connect MICA and HCP5 plus LINC01149. (**bottom**) GeneHancer connections for HCP5 (yellow) and MICA (red) map to predicted enhancers within CNVR1.

We used both GeneHancer (30) (visualized in the UCSC genome browser), which infers genomic connectedness based on multiple genomic data sources, and HiC physical connectedness data (31) to demonstrate genomic interaction between *MICA* and long non-coding regulatory RNAs *HCP5* and *LINC01149* that are both contained within CNVR1 (Fig. 3). This is consistent with a role of these long non-coding RNAs (lncRNAs) in regulating expression of *MICA* within CNVR1.

### Correlation of CNVR1 genotype with serum MICA levels

**Figure 4:**
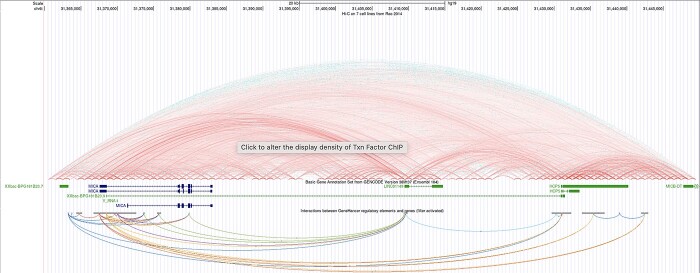
Evaluation of serum levels of soluble MICA. (**A**) Comparison of soluble MICA levels according to the copy number with/without deletions, *P*-value was from one-tailed test. (**B**) Comparisons of soluble MICA levels according to the copy number, *P*-value was from Kruskal–Wallis tests and corrected by false discocery rate. ^*^P ≤ 0.05, ^*^^*^P ≤ 0.01.

CNVR1 encompassed one protein-coding gene, *MICA*. It is a cell-surface receptor for natural killer (NK) T-cells and has a soluble form. Levels of soluble MICA were measured by enzyme-linked immunosorbent assay (ELISA) in 13 individuals with CN = 0, 49 with CN = 1 and 43 with CN = 2, 95% of whom were men. People without the deletion had a statistically significantly increased mean level of circulating MICA protein (Fig. 4; *P* = 0.03) with those of CN = 0 genotype exhibiting the lowest level (55.7 pg/ml), those with CN = 1 an intermediate level (172.9 pg/ml) and those with CN = 2 the highest level (219.9 pg/ml).

## Discussion

We assessed the role of common large (>5 kb) CNV in gout in the Aotearoa NZ Māori and Pacific (Polynesian) populations and identified three common Polynesian-specific CNV regions [Chr6: 31.36–31.45 Mb (CNVR1); Chr1: 196.75–196.92 Mb (CNVR6); Chr1: 189.35–189.54 Mb (CNVR9)] with evidence for association with gout. Additional genetic support for *MICA* as a candidate causal gene in CNVR1 was obtained from European gout genetic association data and from the GTEx genetic control of gene expression resource. However equivalent data were not obtained for either CNVR6 or CNVR9.

Given the limited power of the Polynesian gout sample set, and the unavailability of a replication cohort, our strategy focused on reducing the statistical impact of multiple testing. Hence, we used an approach that would allow us to identify population-specific CNV rather than a genome-wide approach. The European and Han Chinese sample sets were included to serve this purpose. Future studies can focus on the impact of a wider number of common CNV on gout in these populations, leveraging considerably larger sample sizes.

The locus with the strongest evidence for a role in gout was CNVR1, which is dominated by deletion. Genetic (SNV) associations identified by GWAS for immune-mediated disease have been reported at this locus, including HIV infection (32) and autoimmune diseases such as Grave’s disease (33) and systemic lupus erythematosus (34). CNVs that overlap CNVR1 have been reported to be associated with nasopharyngeal carcinoma predisposition (35,36), type 1 diabetes (37) and schizophrenia or bipolar disorder (38) and, now, gout. Recurrent hemizygous deletion at the locus associates with idiopathic pulmonary hemosiderosis, a rare disease without an identified trigger (39). We also provided additional evidence of gout risk in Europeans and gene expression data from GTEx for a role of *MICA* within CNVR1 in the etiology of gout. The CNV encompasses one protein-coding gene (*MICA*), which is a strong candidate causal gene. Levels of the MICA protein are reduced in people with the deletion (Fig. 4). *MICA* encodes a genotoxic stress-induced protein and serves as a ligand for Natural Killer Group 2 member (NKG2D). NKG2D is an immune receptor on the surface of NK cells (40), which binds to immune cells expressing MICA and triggering lysis in these target cells. NK cells can also indirectly activate monocytes and neutrophils through direct interaction or cytokine secretion (41,42). In gout, reduced levels of MICA might result in accumulation of macrophages and an increased immune response to MSU crystals. Urate induces MICA expression via TAK1 promoting NK cell killing and immunosuppression (43–45). Reduction of urate in an animal model via inhibition of xanthine oxidoreductase activity or gene knockout ablates the genotoxic stress-induced expression of MICA. Additionally, genetic variants that associate with MICA serum levels also associate with inflammatory phenotypes giant cell arteritis, ankylosing spondylitis and hepatitis C and also with DNA methylation in the HLA region (46–49). In addition to MICA, three non-protein-coding genes map within *CNVR1*. *HCP5* is a lncRNA gene primarily expressed in the immune system and its expression is modulated in chronic kidney disease (50), allergic rhinitis (51) and multiple cancers (52). *HCP5* could be an example of an immune-priming lncRNA (53) that perhaps modulates expression of *MICA*—we were able to show that within CNVR1 there is physical interaction between the *HCP5* and *MICA* genes via the *LINC01149* locus (Fig. 3). Also mapping within the deletion are pseudogene *HCG26* and non-coding Y RNA ENSG00000199332.1—the functional impact of each is poorly understood. On the basis of current understanding, we speculate that the CNVR1 deletion may increase the risk of gout via perturbed immune activation and surveillance pathways downstream of *MICA* and control of its expression by regulatory RNAs in the locus.

Despite CNVR1 being prevalent in Eastern Polynesian (15.8% in non-gout), it was not associated with gout in this ancestral group for reasons that are, as yet, unclear. However, we have observed a dichotomy in association with gout between Eastern and Western Polynesian at the *ABCG2* locus, despite the gout-associated *rs2231142* risk allele being prevalent in both ancestral groups (54). Similarly, the *ABCG*2 *rs10011796* risk allele was associated with tophi in Western Polynesian but not in Eastern Polynesian people (55). The dichotomy may relate to other interacting variants that also differ in frequency between the Eastern and Western Polynesian ancestral groups. Our results underline that not only pan-ancestry but also sub-regional population differences should be taken into account when conducting biomedical genetic research in the Māori and Pacific populations of Aotearoa NZ.

In conclusion, we identified Polynesian-specific CNVs. Three CNVRs at Chr6: 31.36–31.45 Mb, Chr1: 196.75–196.92 Mb and Chr1: 189.35–189.54 Mb showed evidence for association with gout in Polynesian. We identified *MICA* as a strong candidate causal gene and demonstrated reduced circulating MICA levels in people with deletion at CNVR1. Our study provides new insights into the pathogenic mechanism underlying gout in people of Māori and Pacific ancestry living in Aotearoa NZ.

## Materials and Methods

### Participants

The Aotearoa NZ Polynesian sample set (individuals of Aotearoa NZ and Cook Island Māori, Samoan, Tongan, Niuean and Tokelauan ancestry) comprised 1196 participants with and 1249 without gout (Table 3). All people with gout met the 1977 American Rheumatism Association preliminary gout classification criteria (56). The sample set included 171 Māori with and 98 Māori without gout from the rohe (area) of the Ngāti Porou iwi (tribe) of the Tairāwhiti region of Aotearoa NZ. The Aotearoa NZ European sample set (552 participants without gout) and the Han Chinese sample set (57) (1962 participants without gout) were included for comparison purposes to facilitate identification of Polynesian-specific CNV. All ethnicity was self-reported.

**Table 3:** Age, sex and serum urate details of Polynesian sample sets

Sample set	Classification	Male			Female		
		Number (%)	Age^a^ (SD)	Urate mmol/l^b^ (SD)	Number (%)	Age^a^ (SD)	Urate mmol/l^b^ (SD)
EP	Gout	561(76.6)	56.4 (12.0)	0.40 (0.13)	171 (23.4)	60.7 (12.1)	0.36 (0.14)
	Non-gout	331 (41.6)	45.7 (15.9)	0.38 (0.11)	464 (58.4)	46.7 (15.0)	0.32 (0.05)
WP	Gout	364(86.7)	48.0 (12.3)	0.44 (0.13)	56(13.3)	54.3 (13.5)	0.38 (0.17)
	Non-gout	200 (51.9)	35.5 (14.5)	0.41 (0.10)	185 (48.1)	38.4 (16.6)	0.32 (0.09)
EPWP	Gout	36(81.8)	42.9 (12.9)	0.46 (0.12)	8(18.2)	48.1 (11.0)	0.45 (0.17)
	Non-gout	34 (49.3)	35.1 (17.5)	0.43 (0.09)	35 (50.7)	38.0 (14.5)	0.33 (0.09)

EP, Eastern Polynesian; EPWP, Mixed Eastern–Western Polynesian; WP, Western Polynesian.

^a^Age at diagnosis for gout cases, at recruitment for controls.

^b^Serum urate levels at recruitment. Data presented for individuals for whom sex information was available.

The Polynesian sample set was divided into three groups (Eastern Polynesian, Western Polynesian and Mixed Eastern-Western Polynesian). The Eastern Polynesian sample set composed of Aotearoa NZ Māori, Cook Island Māori, French Polynesian and other Polynesian (excluding Pukapuka) (732 gout, 795 without gout). The Western Polynesian sample set composed of Samoa, Tonga, Tuvalu, Niue, Tokelau, Pukapuka and other Polynesian (420 gout, 385 without gout). The Mixed Eastern–Western Polynesian group composed 44 people with and 69 without gout of mixed Eastern and Western Polynesian ethnicity. Serum urate measurements were performed at the Southern Community Laboratories (www.sclabs.co.nz) for all Aotearoa NZ samples.

### Generation of exome array genotype and WGS Data

A total of 3937 Aotearoa NZ Polynesian and European individuals were genotyped in two batches (batch 1 with 291 and batch 2 with 3646 individuals) on the Illumina Infinium CoreExome-24 platform. (2997 used in this study were a subset.) From the data generated, log R ratios (LRRs) and B allele frequencies (BAFs) were extracted from the autosomes and used to identify CNV. Samples with a genotyping call rate of <98% and SNVs with a call rate of <95% were not utilized in the subsequent analysis (58). Principal component (PC) analysis was applied to the LRR values, and the first six PCs were visualized in scatter plots to evaluate the possibility of batch effects. Differential effects were not identified when samples were classified according to batch (Supplementary Material, Fig. S7) or ancestral group (Supplementary Material, Fig. S8). The Han Chinese controls of 1962 individuals were genotyped using the Affymetrix axiom genome-wide Chinese Han Beijiing array. Samples with a genotyping call rate of <95% and SNVs with a call rate of <95% were excluded. A total of 1848 Han Chinese samples were used in the subsequent analyses. Ninety-one Aotearoa NZ Polynesian individuals were sequenced to high coverage on a HiSeqX machine using TruSeq Nano libraries to allow validation of identified CNVRs.

### CNV identification and assignment of genotype

Supplementary Material, Figure S9 illustrates the workflow. All genomic locations were derived from NCBI GRCh37/UCSC hg19 coordinates. LRR and BAF values were used to determine copy number. As a normalized measure of total signal intensity, LRR values of 0 represent two copies with lower values in identifying areas of loss and higher values areas of gain. The BAF is a measure of the allelic intensity ratio, with values ranging from 0 to 1. Areas of homozygosity (e.g. deletion) have BAF of 0 or 1, normal diploid regions have BAF of 0, 0.5 or 1 and allelic imbalance areas (e.g. duplication) show intermediate values. PennCNV (v1.0.5) (59) was applied to identify CNV (CN ≠ 2), including homozygous deletion, heterozygous deletion and duplication. This tool optimized a trained hidden Markov model transition file and a guanine–cytosine (GC) content file that was created for all the SNVs to segment the normalized and transformed probe intensities (LRRs and BAFs) and incorporate population SNV frequencies of the B allele (PFB). In this study, the PFB values for all SNVs of each group were computed from the relevant non-gout sample sets via their intensity files and the ‘compile_pfb.pl’ script in PennCNV. Then, CNV calls were generated using the ‘detect_cnv.pl’ script, along with the customized parameter files (PFB files and GC content files) and intensity files.

### CNV quality control and definition of CNVR

Any CNVs that were supported by <10 SNPs or were <5 kb in length and were low confidence (confidence score <40) were removed (Supplementary Material, Fig. S9). Additional subject quality control was performed to exclude subjects: samples with LRR standard deviation >0.28, a waviness factor (the amount of dispersion in signal intensity) (60) >0.05 or < −0.05, or CNV count >312 (85th percentile, see Supplementary Material, Fig. S10) were not utilized in downstream analyses (61). Subsequently, all CNV calls were manually confirmed by visualizing LRR and BAF plots. When overlapping CNVs were identified, they were merged into unique CNV regions via CNVRuler (62), taking the outmost boundaries of the union of those CNVs (Supplementary Material, Table S1).

### Statistical analysis

All statistical analysis was performed using R (version 4.0.3). To test for statistically significant differences in the distribution of copy number between cases and controls, regression analyses were implemented within each Polynesian population group. Two models were used: adjustment with age and sex (Model 1); and Model 1 plus adjustment by genotyping batches and PCs 1–10 derived from the genome-wide genotype data as previously described (29) (Model 2). Combination of the three Polynesian subsets was done by meta-analysis with fixed-effects model (except for CNVR1 and burden test). Kruskal–Wallis test and Mann–Whitney test were used to compare serum MICA levels between groups. A *P* < 0.05 threshold indicated nominal evidence for association.

### Validation of CNV calls in WGS and publicly available datasets

Among the Polynesian cohort, 91 individuals with both microarray and WGS data were used to confirm the presence of CNV calls. Each chromosome was divided into 1 kb bins, and read depth of coverage in each bin was calculated by Samtools (63). The read depth of the bins was plotted to confirm the deletions and duplications. The DGV (http://dgv.tcag.ca/dgv/app/home) that includes annotated CNVs was also used for confirmation of CNV.

### Gout GWAS in UK Biobank

A total of 332 370 European individuals from the UK Biobank (64) were included under the approval number 12611. The UK Biobank has ethical approval from the North West Multi-Centre Research Ethics Committee (11/NW/0382) and obtained written informed consent from all participants prior to the study. Gout cases were defined as individuals with any of self-reported gout, urate-lowering therapy use (allopurinol or sulfinpyrazone) with no diagnosis of leukemia or lymphoma (ICD codes C81 and C96), primary or secondary diagnosis of gout (ICD code M10) (11). Exclusion criteria were sex chromosome and self-reported sex mismatch, genotype QC failure, relatedness (KING coefficient >0.177) and primary or secondary kidney disease. The GWAS was done using a total of 27 287 012 variants imputed from 845 487 genotyped variants. A logistic regression model was produced for each variant adjusting for age, sex and the first 40 genetic PCs using Plink version 1.9 6.10 (65).

### Enzyme-linked immunosorbent assay

A Human MICA ELISA Kit (Invitrogen, Thermo Fisher Scientific, Waltham, Maryland, USA) was used to detect soluble MICA (following the manufacturer’s instructions) in serum that had been stored at −80°C. Absorbance was measured at 450 nm. A standard curve of the logarithmic relationship between concentration and absorbance was used to calculate the concentration of soluble MICA in serum samples. All people homozygous for the deletion (CN = 0) were selected for measuring of MICA serum levels. For each of CN = 1 and CN = 2, we began by randomly selecting 30 gout and 30 non-gout individuals (i.e. 60 for each genotype). However, after accounting for unavailability of serum or insufficient serum, the eventual numbers assayed were 49 for CN = 1 and 43 for CN = 2. Association data across the CNVR1, CNVR6 and CNVR9 were specifically queried from these GWAS data.

## Author contributions

T.R.M., Y.S. and K.W. designed and supervised the project. K.W., M.C., M.B., M.P.L., M.E.M., Q.Y., Z.L., R.T., A.P.-G., T.J.M, R.T., N.D., F.K., R.M., L.K.S, J.d.Z. and Z.W collected and contributed to analysis of data. K.W. and T.R.M wrote the manuscript with input from co-authors. All authors contributed to and have approved the final manuscript.

## Supplementary Material

PolynesianCNV_supplement_material_coauthors_1208_revised_ddac094

Table_S3_ddac094
