# Supplementary material for: A Polynesian-specific copy number variant encompassing the MICA gene associates with gout
Source: Hum Mol Genet. 2022 Apr 22;31(21):3757–68. doi: 10.1093/hmg/ddac094 (PMC9616569; doi:10.1093/hmg/ddac094)

**SUPPLEMENTAL MATERIAL**

**A Polynesian-specific copy number variant encompassing the MHC Class I Polypeptide-related Sequence A (MICA) gene associates with gout**

Ke Wang^1,2^, Murray Cadzow^2^, Matt Bixley^2^, Marilyn E. Merriman^2^, Qiangzhen Yang^1^, Zhiqiang Li^1,4^, Riku Takei^2,3^, Amanda Phipps-Green^2^, Tanya J. Major^2^, Ruth Topless^2^, Nicola Dalbeth^5^, Frances King^6^, Rinki Murphy^5^, Lisa K. Stamp^7^, Janak de Zoysa^5^, Zhuo Wang^1^, Yongyong Shi ^1,4, *^ & Tony R. Merriman^2,3, *^

^1^ Bio-X Institutes, Key Laboratory for the Genetics of Developmental and Neuropsychiatric Disorders (Ministry of Education), Collaborative Innovation Center for Brain Science, Shanghai Jiao Tong University, Shanghai 200030, People’s Republic of China

^2^ Department of Biochemistry, University of Otago, Dunedin, New Zealand

^3^ Division of Clinical Immunology and Rheumatology, University of Alabama at Birmingham, Birmingham, Alabama, United States

^4^ Biomedical Sciences Institute of Qingdao University (Qingdao Branch of SJTU Bio-X Institutes), Qingdao University, Qingdao, 266003, China

^5^ Department of Medicine, University of Auckland, Auckland, New Zealand

^6^ Ngati Porou Hauora Charitable Trust, Te Puia Springs, New Zealand

^7^ Department of Medicine, University of Otago, Christchurch, New Zealand

^*^Corresponding authors

| Table S1. CNVR boundary and genotype frequency information. | | | | | | | | | | | | | |
| --- | --- | --- | --- | --- | --- | --- | --- | --- | --- | --- | --- | --- | --- |
| **CNVR** | **Chr** | **Start (Mb)** | **End (Mb)** | **Type** | **Counts** | **Copy Number** | | | | **Prevalence** | | | |
|  |  |  |  |  |  | **CN=0** | **CN=1** | **CN=3** | **CN=4** | **POLY gout** | **POLY**  **non-gout** | **EUR non-gout** | **CHN non-gout** |
| CNVR1 | chr6 | 31360255 | 31453618 | mixed | 347 | 26(0.57%) | 304(9.08%) | 17(0.51%) | 0(0%) | 17.6% | 14.9% | 0.0% | 0.0% |
| CNVR2 | chr6 | 29918099 | 29937833 | mixed | 278 | 28(0.84%) | 238(7.11%) | 12(0.36%) | 0(0%) | 13.2% | 13.9% | 0.0% | 0.0% |
| CNVR3 | chr10 | 47543322 | 47703946 | mixed | 158 | 0(0%) | 8(0.24%) | 150(4.48%) | 0(0%) | 1.5% | 2.5% | 7.8% | 0.0% |
| CNVR4 | chr12 | 31256795 | 31410284 | mixed | 137 | 0(0%) | 4(0.12%) | 133(3.97%) | 0(0%) | 1.5% | 1.5% | 10.8% | 0.0% |
| CNVR5 | chr12 | 8003758 | 8123306 | mixed | 120 | 0(0%) | 17(0.51%) | 103(3.08%) | 0(0%) | 4.0% | 3.8% | 3.2% | 0.1% |
| CNVR6 | chr1 | 196748331 | 196918615 | loss | 117 | 50(1.49%) | 67(2%) | 0(0%) | 0(0%) | 6.6% | 4.2% | 0.0% | 0.0% |
| CNVR7 | chr2 | 110863908 | 110983320 | mixed | 109 | 0(0%) | 12(0.36%) | 97(2.9%) | 0(0%) | 5.6% | 3.6% | 0.5% | 0.0% |
| CNVR8 | chr13 | 111167417 | 111330405 | mixed | 98 | 0(0%) | 2(0.06%) | 96(2.84%) | 0(0%) | 5.0% | 4.4% | 0.0% | 0.0% |
| CNVR9 | chr1 | 189350700 | 189542729 | mixed | 93 | 3(0.09%) | 89(2.66%) | 1(0.03%) | 0(0%) | 5.3% | 3.6% | 0.0% | 0.0% |
| CNVR10 | chr10 | 135256762 | 135379710 | mixed | 74 | 0(0%) | 2(0.06%) | 72(2.15%) | 0(0%) | 1.9% | 1.5% | 2.3% | 0.0% |
| CNVR11 | chr20 | 58440438 | 58502456 | mixed | 66 | 0(0%) | 65(1.82%) | 1(0.03%) | 0(0%) | 0.3% | 2.2% | 2.8% | 0.0% |
| CNVR12 | chr22 | 25658525 | 25910879 | mixed | 65 | 0(0%) | 20(0.6%) | 45(1.34%) | 0(0%) | 0.9% | 1.1% | 4.1% | 0.0% |
| CNVR13 | chr6 | 168343838 | 168599333 | gain | 59 | 0(0%) | 0(0%) | 59(1.7%) | 0(0%) | 0.7% | 0.4% | 3.5% | 0.0% |
| CNVR14 | chr8 | 137688230 | 137852251 | loss | 54 | 1(0.03%) | 53(1.58%) | 0(0%) | 0(0%) | 0.5% | 0.6% | 3.5% | 0.0% |
| CNVR15 | chr14 | 62634580 | 62767872 | gain | 51 | 0(0%) | 0(0%) | 51(1.52%) | 0(0%) | 2.7% | 2.2% | 0.0% | 0.0% |
| CNVR16 | chr5 | 121844320 | 122051019 | gain | 34 | 0(0%) | 0(0%) | 34(1.02%) | 0(0%) | 1.7% | 1.5% | 0.0% | 0.0% |
| CNVR17 | chr17 | 44169808 | 44293020 | gain | 38 | 0(0%) | 0(0%) | 38(1.14%) | 0(0%) | 0.1% | 0.0% | 0.9% | 0.0% |
| CNVR18 | chr22 | 18801657 | 19016663 | mixed | 37 | 0(0%) | 5(0.15%) | 32(0.93%) | 0(0%) | 0.8% | 0.7% | 2.3% | 0.0% |
| CNVR19 | chr3 | 151514590 | 151545958 | loss | 35 | 0(0%) | 35(1.05%) | 0(0%) | 0(0%) | 0.4% | 0.5% | 1.2% | 0.0% |
| CNVR20 | chr20 | 25434092 | 25581424 | mixed | 29 | 0(0%) | 3(0.09%) | 26(0.75%) | 0(0%) | 1.7% | 0.9% | 0.0% | 0.0% |
| CNVR21 | chr7 | 153485627 | 153670448 | gain | 32 | 0(0%) | 0(0%) | 32(0.96%) | 0(0%) | 1.6% | 1.5% | 0.0% | 0.0% |
| CNVR22 | chr7 | 76131646 | 76639871 | mixed | 31 | 0(0%) | 10(0.3%) | 21(0.63%) | 0(0%) | 0.5% | 0.9% | 1.4% | 1.3% |
| CNVR23 | chr19 | 43372386 | 43766659 | mixed | 28 | 1(0.03%) | 17(0.51%) | 10(0.3%) | 0(0%) | 0.7% | 0.7% | 0.7% | 0.0% |
| CNVR24 | chr12 | 63946056 | 64151350 | mixed | 26 | 0(0%) | 5(0.15%) | 21(0.63%) | 0(0%) | 0.3% | 0.5% | 1.6% | 0.0% |
| CNVR25 | chr15 | 24357212 | 24668007 | mixed | 24 | 1(0.03%) | 21(0.63%) | 2(0.06%) | 0(0%) | 1.0% | 0.6% | 0.5% | 0.1% |
| CNVR26 | chr7 | 149551993 | 149806645 | gain | 22 | 0(0%) | 0(0%) | 22(0.66%) | 0(0%) | 1.3% | 0.8% | 0.0% | 0.0% |
| CNVR27 | chr11 | 134353827 | 134715777 | mixed | 22 | 0(0%) | 11(0.33%) | 11(0.33%) | 0(0%) | 1.0% | 0.2% | 0.5% | 0.1% |
| CNVR28 | chr10 | 45218841 | 45359483 | gain | 21 | 0(0%) | 0(0%) | 21(0.63%) | 0(0%) | 0.2% | 0.1% | 1.2% | 0.0% |
| CNVR29 | chr18 | 7080306 | 7562317 | gain | 20 | 0(0%) | 0(0%) | 20(0.6%) | 0(0%) | 0.8% | 1.0% | 0.0% | 0.0% |
| CNVR30 | chr2 | 242873800 | 243034519 | loss | 19 | 3(0.09%) | 16(0.48%) | 0(0%) | 0(0%) | 0.1% | 0.4% | 0.9% | 0.0% |
| CNVR31 | chr9 | 11937390 | 12216756 | mixed | 19 | 2(0.06%) | 16(0.48%) | 1(0.03%) | 0(0%) | 0.8% | 0.7% | 0.0% | 0.0% |
| CNVR32 | chr18 | 1913316 | 1972346 | mixed | 18 | 0(0%) | 17(0.51%) | 1(0.03%) | 0(0%) | 0.3% | 0.1% | 1.4% | 0.0% |
| CNVR33 | chr16 | 15125627 | 16301530 | mixed | 17 | 0(0%) | 1(0.03%) | 16(0.45%) | 0(0%) | 0.6% | 0.6% | 0.0% | 0.0% |
| CNVR34 | chr4 | 92225277 | 93428415 | mixed | 16 | 0(0%) | 1(0.03%) | 15(0.45%) | 0(0%) | 0.9% | 0.5% | 0.2% | 0.0% |
| CNVR35 | chr4 | 2172826 | 2252881 | loss | 15 | 0(0%) | 15(0.45%) | 0(0%) | 0(0%) | 0.0% | 0.3% | 0.2% | 0.0% |
| CNVR36 | chr12 | 19469320 | 19578107 | gain | 15 | 0(0%) | 0(0%) | 15(0.45%) | 0(0%) | 0.3% | 0.2% | 0.7% | 0.0% |
| CNVR37 | chr14 | 20213937 | 20416499 | mixed | 15 | 0(0%) | 1(0.03%) | 14(0.42%) | 0(0%) | 0.3% | 0.4% | 0.0% | 0.0% |
| CNVR38 | chr9 | 138149166 | 138302726 | gain | 14 | 0(0%) | 0(0%) | 14(0.42%) | 0(0%) | 0.0% | 0.0% | 1.2% | 0.0% |
| CNVR39 | chr15 | 61939189 | 62182599 | gain | 14 | 0(0%) | 0(0%) | 14(0.42%) | 0(0%) | 0.5% | 0.9% | 0.0% | 0.0% |
| CNVR40 | chr20 | 14553745 | 15602064 | loss | 14 | 0(0%) | 14(0.42%) | 0(0%) | 0(0%) | 0.3% | 0.1% | 0.7% | 2.1% |
| CNVR41 | chr7 | 64566009 | 65089380 | mixed | 13 | 0(0%) | 6(0.18%) | 7(0.21%) | 0(0%) | 0.5% | 0.0% | 0.5% | 0.0% |
| CNVR42 | chr19 | 53910518 | 54010277 | mixed | 13 | 0(0%) | 4(0.12%) | 9(0.27%) | 0(0%) | 0.2% | 0.3% | 0.7% | 0.2% |
| CNVR43 | chr15 | 32049125 | 32514341 | gain | 12 | 0(0%) | 0(0%) | 12(0.36%) | 0(0%) | 0.2% | 0.0% | 0.9% | 0.1% |
| CNVR44 | chr12 | 31997981 | 32056577 | gain | 11 | 0(0%) | 0(0%) | 11(0.33%) | 0(0%) | 0.2% | 0.3% | 0.2% | 0.0% |
| CNVR45 | chr22 | 22312879 | 22569035 | mixed | 11 | 0(0%) | 1(0.03%) | 10(0.3%) | 0(0%) | 0.2% | 0.0% | 0.9% | 0.0% |
| CNVR46 | chr1 | 1386089 | 1431163 | loss | 10 | 0(0%) | 10(0.3%) | 0(0%) | 0(0%) | 0.0% | 0.1% | 1.2% | 0.0% |
| CNVR47 | chr3 | 565961 | 2621687 | mixed | 10 | 0(0%) | 2(0.06%) | 8(0.24%) | 0(0%) | 0.1% | 0.2% | 0.7% | 1.8% |
| CNVR48 | chr3 | 100351696 | 100427937 | gain | 10 | 0(0%) | 0(0%) | 10(0.3%) | 0(0%) | 0.0% | 0.1% | 0.9% | 0.0% |
| CNVR49 | chr6 | 61967504 | 62362867 | gain | 10 | 0(0%) | 0(0%) | 9(0.27%) | 1(0.03%) | 0.6% | 0.2% | 0.2% | 0.0% |
| CNVR50 | chr7 | 61852895 | 62840776 | mixed | 10 | 0(0%) | 1(0.03%) | 9(0.27%) | 0(0%) | 0.1% | 0.3% | 0.2% | 0.0% |
| CNVR51 | chr15 | 22770994 | 23236972 | mixed | 10 | 0(0%) | 2(0.06%) | 8(0.21%) | 0(0%) | 0.1% | 0.1% | 0.9% | 0.0% |
| CNVR52 | chr18 | 1731154 | 1841469 | loss | 10 | 0(0%) | 10(0.3%) | 0(0%) | 0(0%) | 0.2% | 0.0% | 0.7% | 0.0% |
| CNVR53 | chr18 | 65848177 | 65899000 | loss | 10 | 0(0%) | 10(0.3%) | 0(0%) | 0(0%) | 0.2% | 0.1% | 0.2% | 0.0% |
| CNVR54 | chr22 | 16855618 | 17544834 | gain | 10 | 0(0%) | 0(0%) | 10(0.3%) | 0(0%) | 0.1% | 0.3% | 0.9% | 0.0% |

POLY, Polynesian; EUR, European; CHN, Chinese. This table only shows those CNVs that were identified in 10 or more individuals. The prevalence represents the proportion of people with CN≠2 in each sample-set. Note that some individuals have more than one CNV in a classified CNV boundary.

| Table S2. Linear regression association analysis with serum urate level in each control cohort | | | | | | | | | |
| --- | --- | --- | --- | --- | --- | --- | --- | --- | --- |
| **Region** | **Ethnicity** | **Sample size** | **Unadjusted** | | **Adjusted (Model1)** | | | **Adjusted (Model2)** | |
|  |  |  | **Beta(95%CI)** | ***P*** | **Beta(95%CI)** | ***P*** | | **Beta(95%CI)** | ***P*** |
| CNVR1 | WP | 267 | 0.01(-0.03-0.04) | 0.81 | 0(-0.04-0.03) | 0.94 | | 0.01(-0.03-0.04) | 0.65 |
|  | EP | 637 | 0.02(0-0.04) | 0.08 | 0.02(0-0.04) | 0.06 | | 0.02(0-0.04) | 0.1 |
|  | EPWP | 55 | 0(-0.13-0.12) | 0.94 | 0.01(-0.10-0.12) | 0.88 | | 0.04(-0.07-0.15) | 0.47 |
|  | OP-meta | 959 | 0.01(-0.01-0.03) | 0.17 | 0.01(-0.01-0.03) | 0.2 | | 0.02(0-0.04) | 0.06 |
|  |  |  |  | *P_het_*=0.65**,** I^2^=0% |  | *P_het_*=0.39**,** I^2^=0% | |  | *P_het_*=0.85**,** I^2^=0% |
| CNVR6 | WP | 267 | 0(-0.05-0.04) | 0.94 | 0(-0.05-0.03) | 0.69 | | 0(-0.04-0.04) | 0.87 |
|  | EP | 637 | -0.03(-0.08-0.03) | 0.38 | -0.03(-0.08-0.03) | 0.37 | | -0.03(-0.08-0.03) | 0.35 |
|  | EPWP | 55 | 0.04(-0.10-0.19) | 0.55 | -0.01(-0.15-0.12) | 0.85 | | -0.02(-0.16-0.13) | 0.83 |
|  | OP-meta | 959 | 0.05(0-0.11) | 0.07 | -0.02(-0.05-0.01) | 0.28 | | -0.01(-0.05-0.02) | 0.45 |
|  |  |  |  | *P_het_*=0.13**,** I^2^=51% |  | *P_het_*=0.85**,** I^2^=0% | |  | *P_het_*=0.65**,** I^2^=0% |
| CNVR9 | WP | 267 | 0(-0.07-0.07) | 0.98 | -0.02(-0.08-0.05) | 0.58 | | 0(-0.07-0.05) | 0.77 |
|  | EP | 637 | 0(-0.04-0.05) | 0.8 | 0(-0.04-0.05) | 0.81 | | 0(-0.04-0.04) | 0.98 |
|  | EPWP | 55 | -0.12(-0.32-0.08) | 0.25 | -0.05(-0.25-0.12) | 0.5 | | -0.07(-0.25-0.12) | 0.48 |
|  | OP-meta | 959 | 0(-0.04-0.03) | 0.83 | -0.01(-0.04-0.03) | 0.67 | | -0.01(-0.04-0.03) | 0.66 |
|  |  |  |  | *P_het_*=0.48**,** I^2^=0% |  | *P_het_*=0.74**,** I^2^=0% | |  | *P_het_*=0.71**,** I^2^=0% |
| EP, Eastern Polynesian; EPWP, Mixed Eastern-Western Polynesian; WP, Western Polynesian; OP-meta, WP, EP and EPWP were combined by meta-analysis; CI, confidence intervals; Model 1, adjusted by age and sex; Model 2, Model 1 plus adjustment by batches and PCs 1-10. | | | | | | |  |  |  |

**Table S3** Database of Genomic Variants (DGC)

Refer separate Excel file,

Table S4. CNV validation: concordance between copy number genotype calculated using WGS and microarray data in 91 Aotearoa NZ Polynesian individuals.

| **Issue** | **Number** | | |
| --- | --- | --- | --- |
|  | **CNVR1** | **CNVR6** | **CNVR9** |
| without deletion in both microarray and WGS | 80 | 66 | 80 |
| with deletion in both microarray and WGS | 7 | 14 | 8 |
| with deletion in microarray, without deletion in WGS | 0 | 0 | 0 |
| without deletion in microarray, with deletion in WGS | 4 | 11 | 3 |
| Percentage genotype agreement | 95.6% | 87.9% | 96.7% |

Figure S1. LRR and BAF plots for very large CNVs.

(A) CNV (chr8:2094501-146245371, ~144Mb, CN=3); (B) CNV (chr15:45571209 -102398631, ~57Mb; chr15:31526389-43478511, ~12Mb, CN=3).


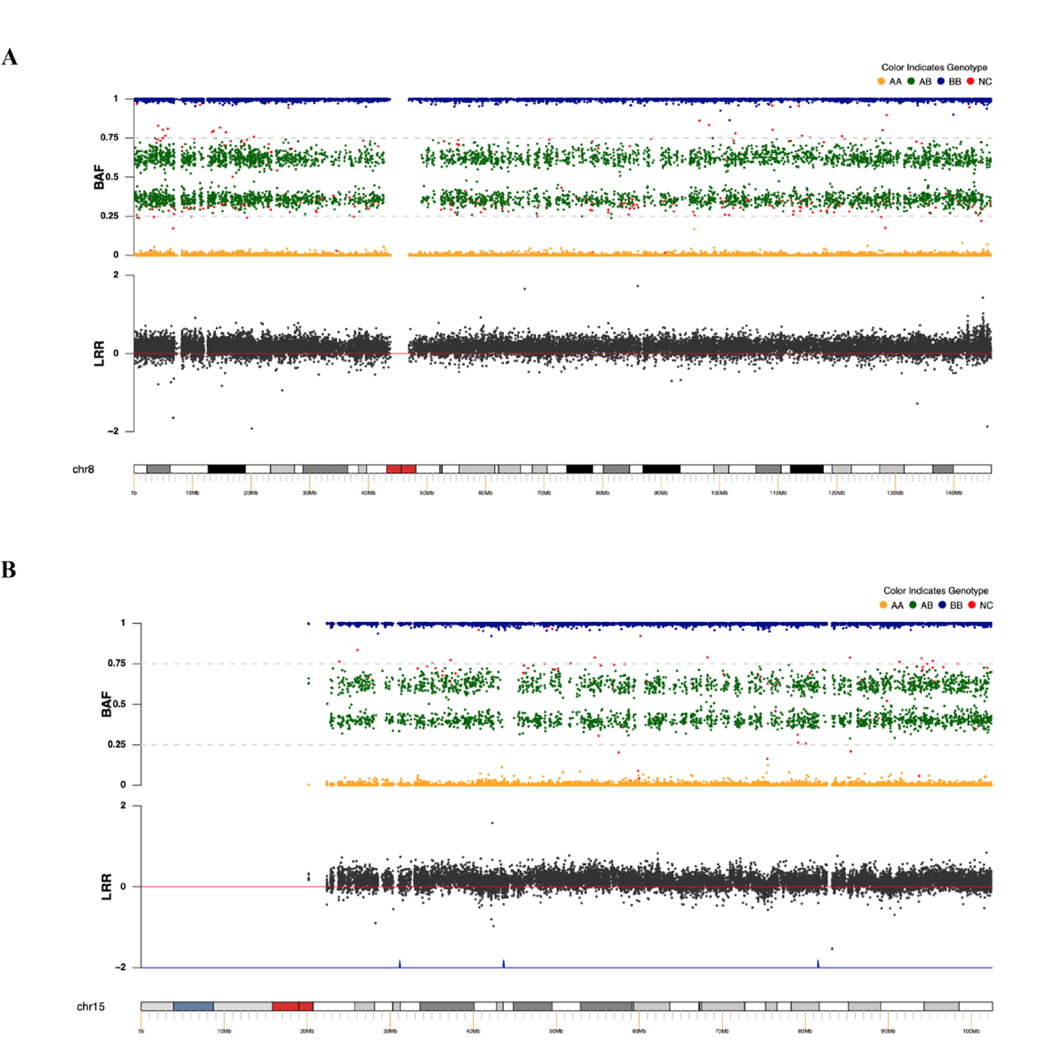


Figure S2. Representative examples of LRR and BAF plots for the 20 most common CNVRs.


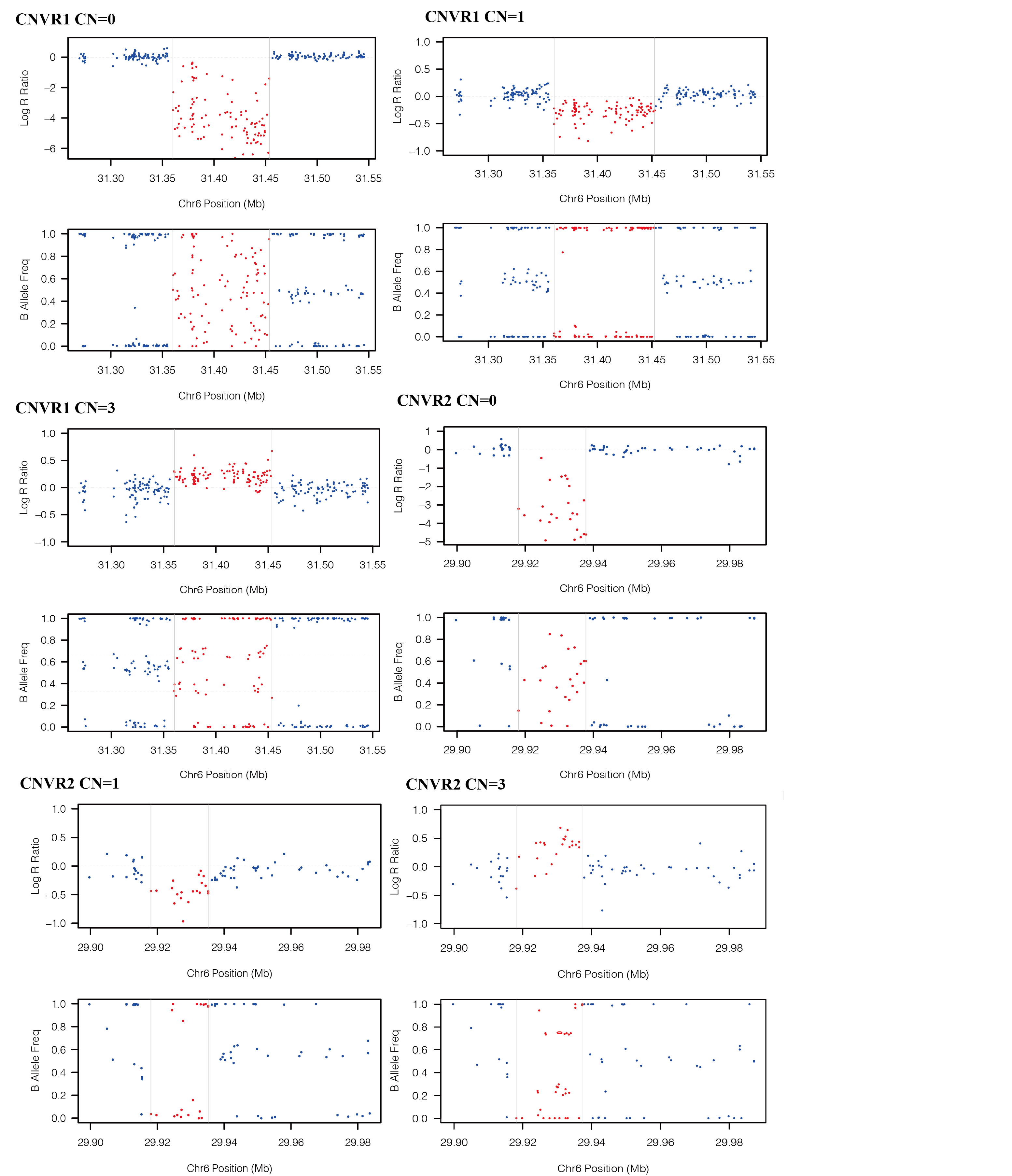


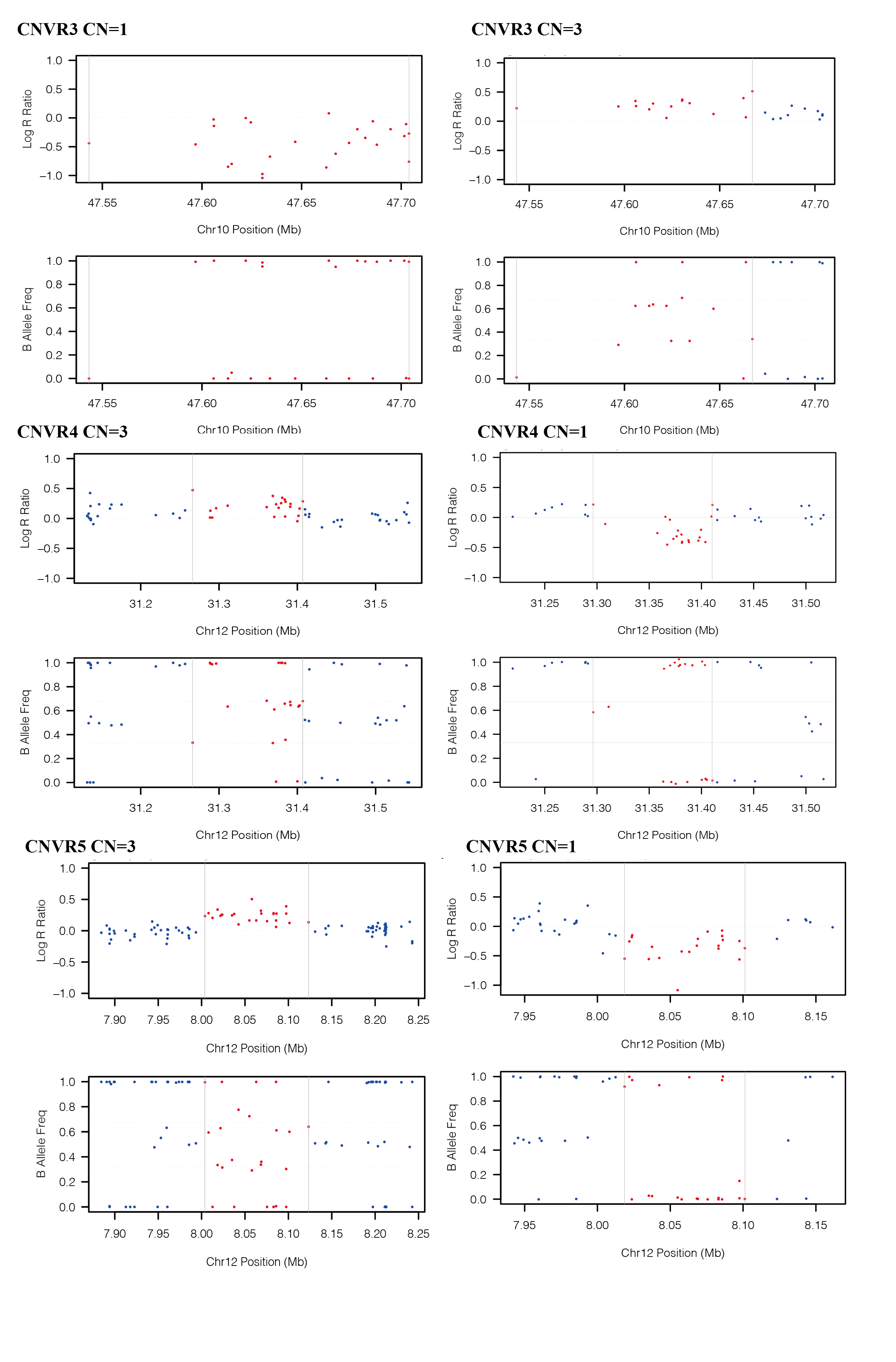


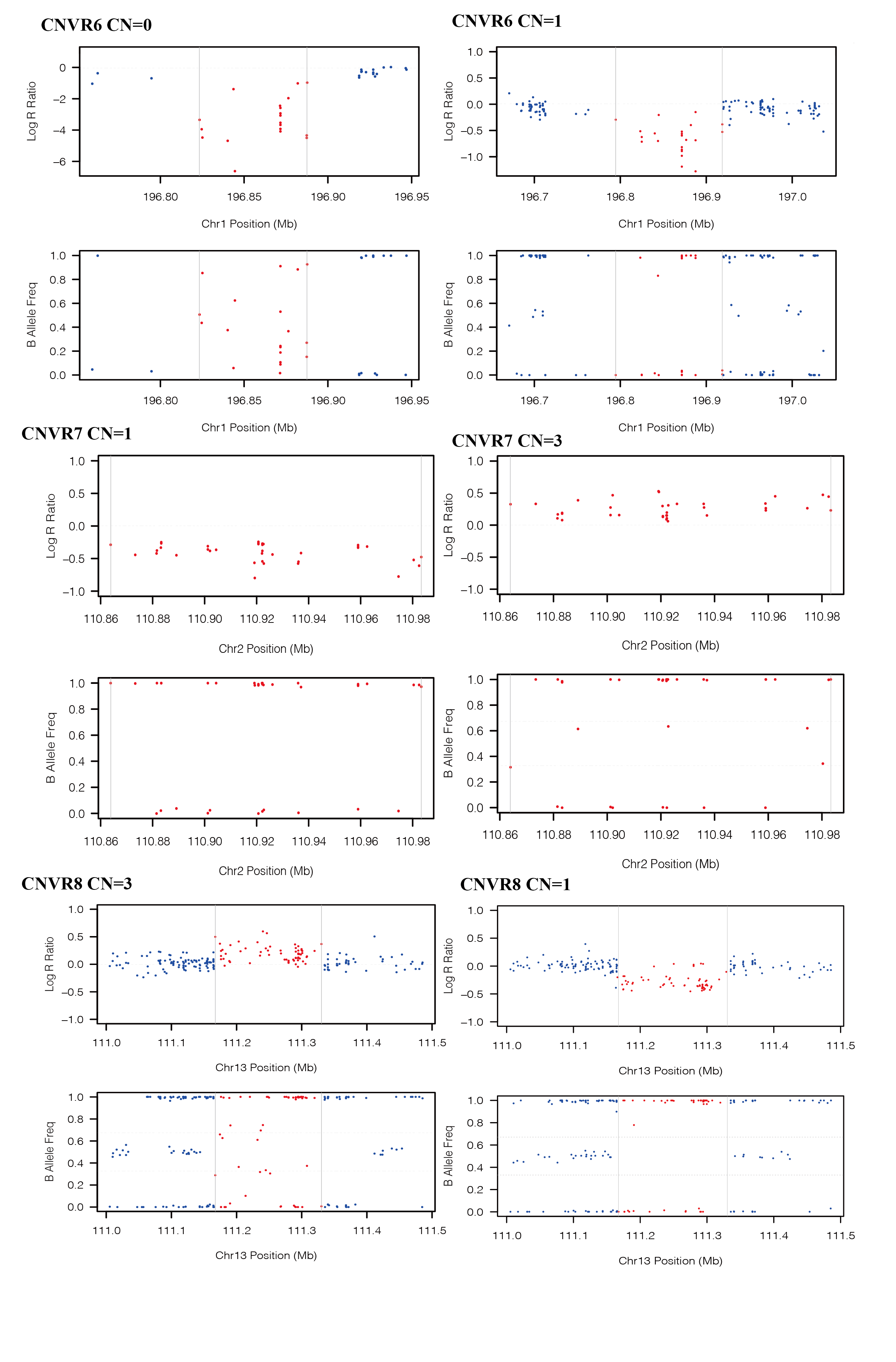

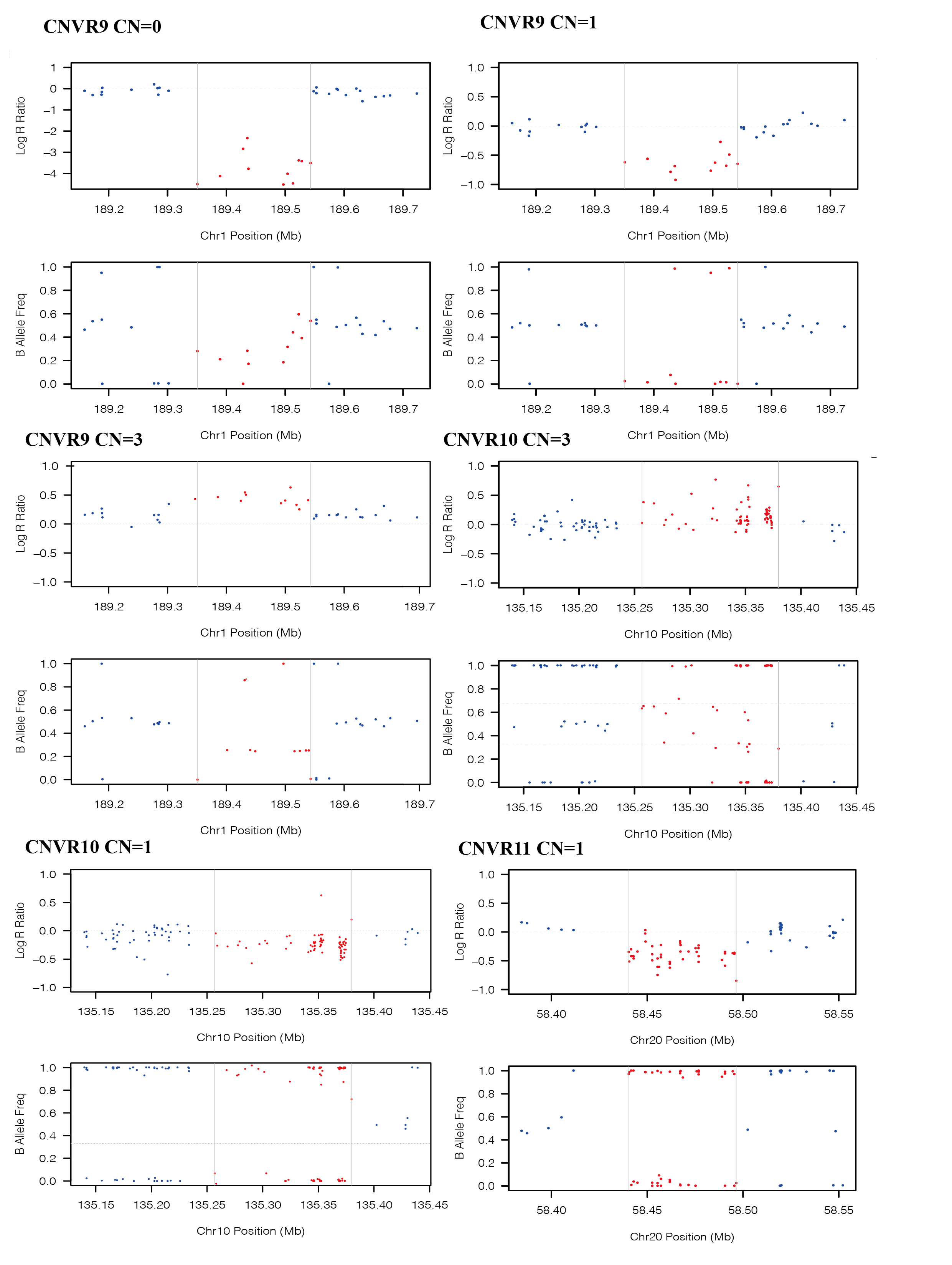

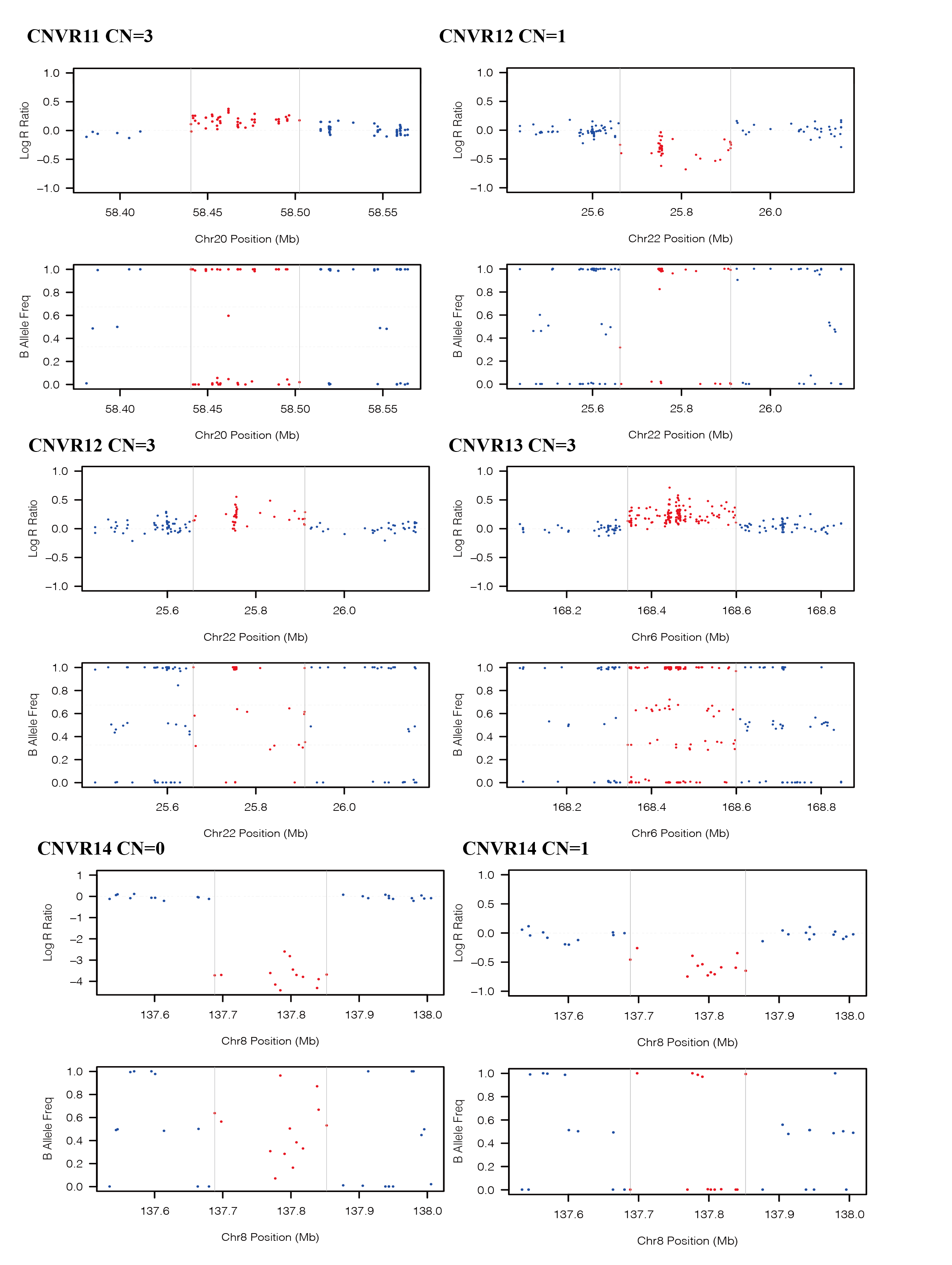

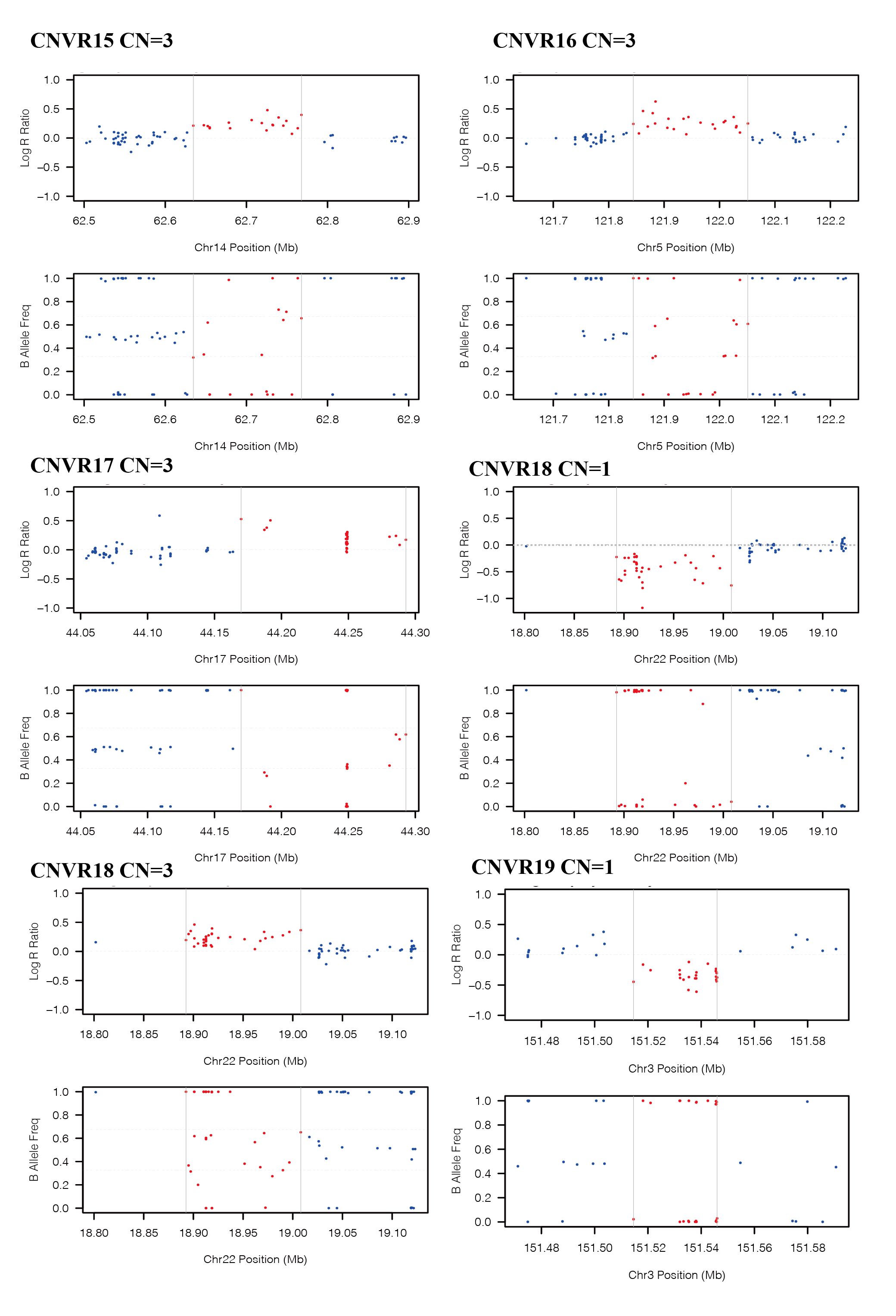

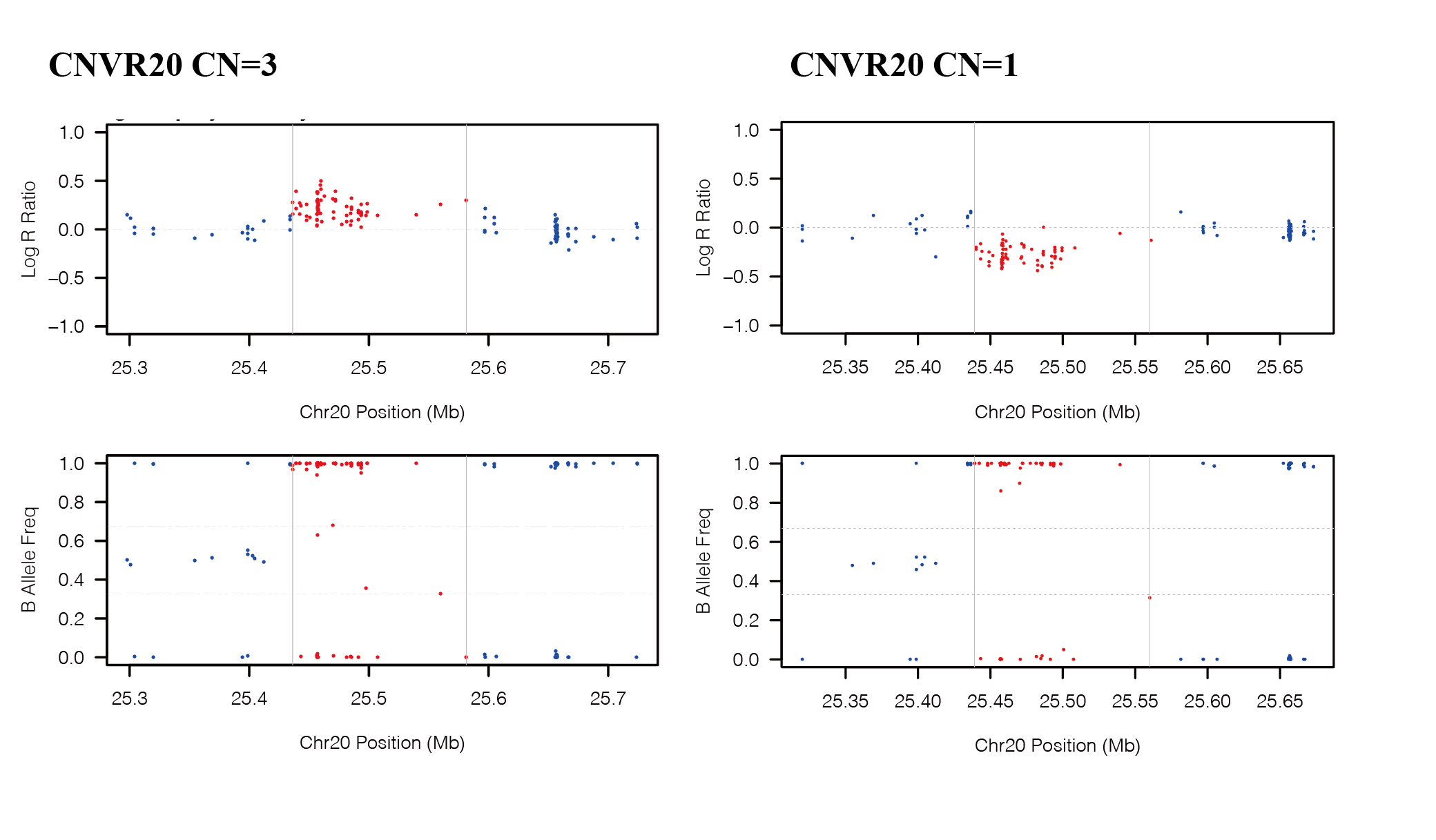


Figure S3. Pairwise comparison of CNV length and genotype count between gout and controls in the Aotearoa NZ Polynesian sample-set.

(A) Length of all classified CNVs (bp, base pair), violin plots. (B) Boxplot comparing CN≠2 genotype counts between people with and without gout.


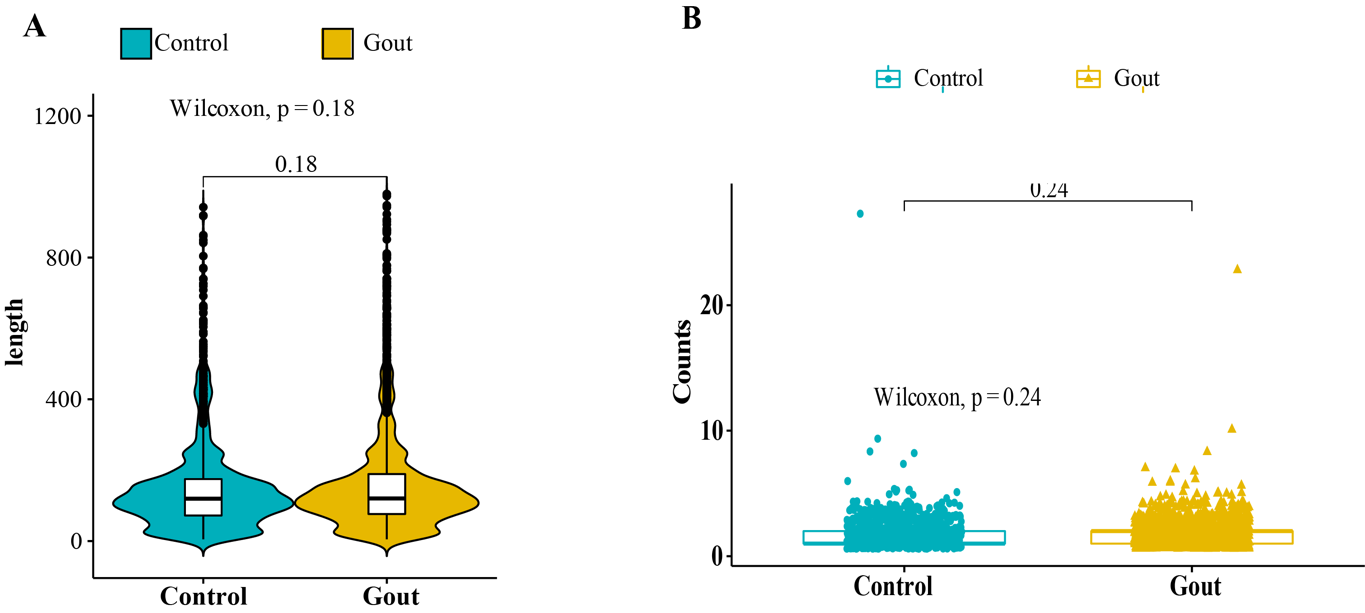


Figure S4. Validation CNVRs in WGS data.

Each chromosome was divided into 1kb bins, and read depth of coverage in each bin was calculated. (A) two representative plots of CNVR1; (B) two representative plots of CNVR6; (C) two representative plots of CNVR9.


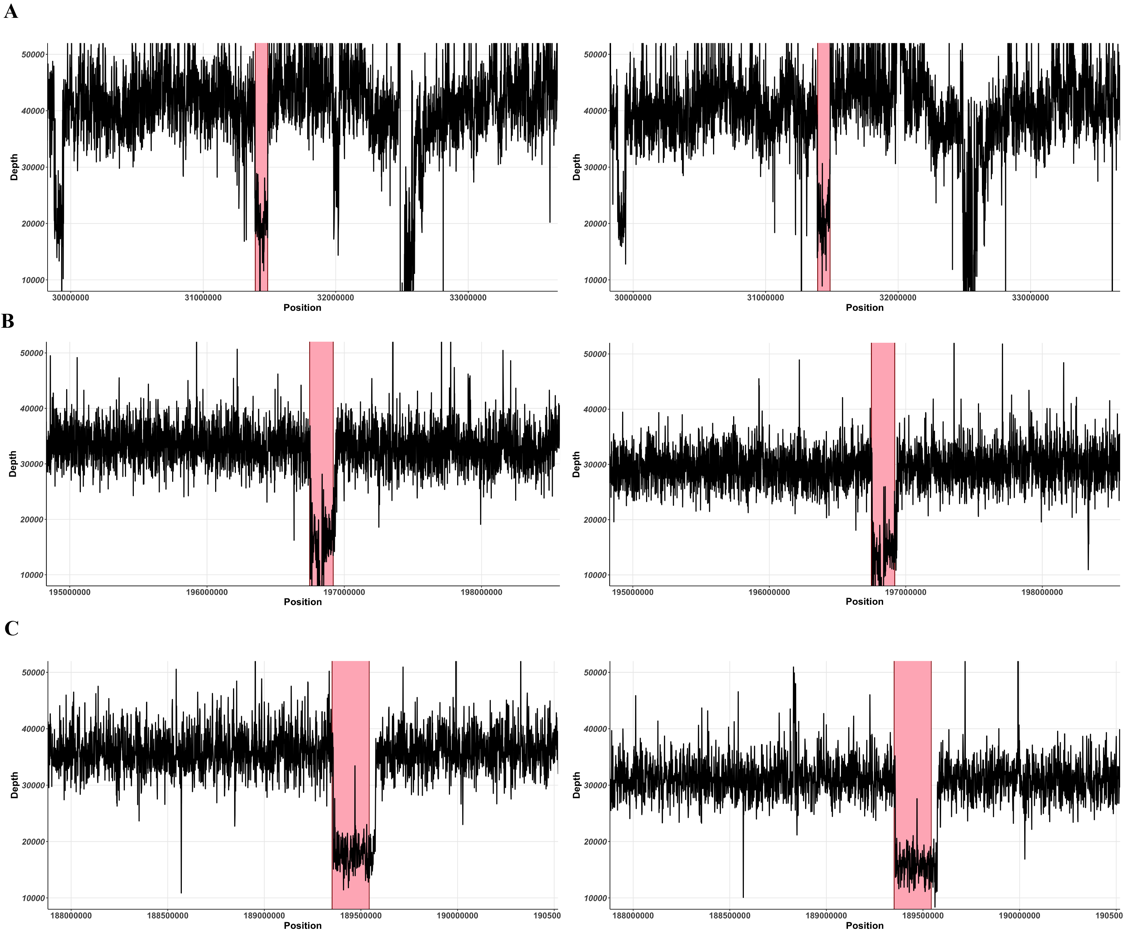


**Figure S5** Association of CNVR1, CNVR6 and CNVR9 with gout in the UK Biobank.


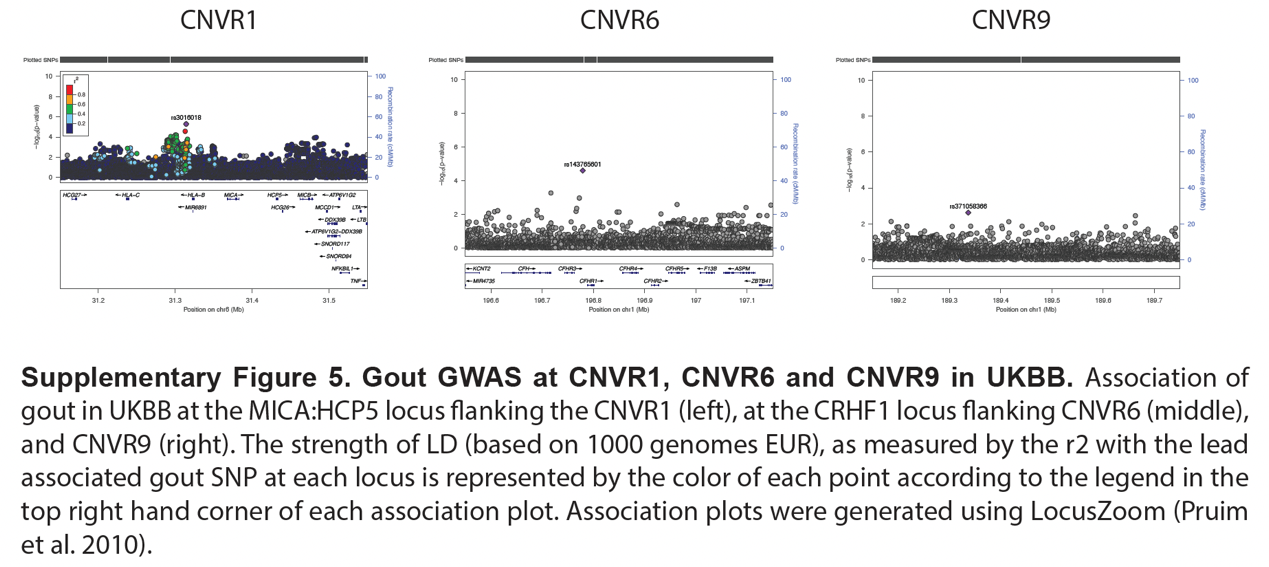


**Figure S6.** Gout GWAS and eQTL for *MICA* at CNVR1.


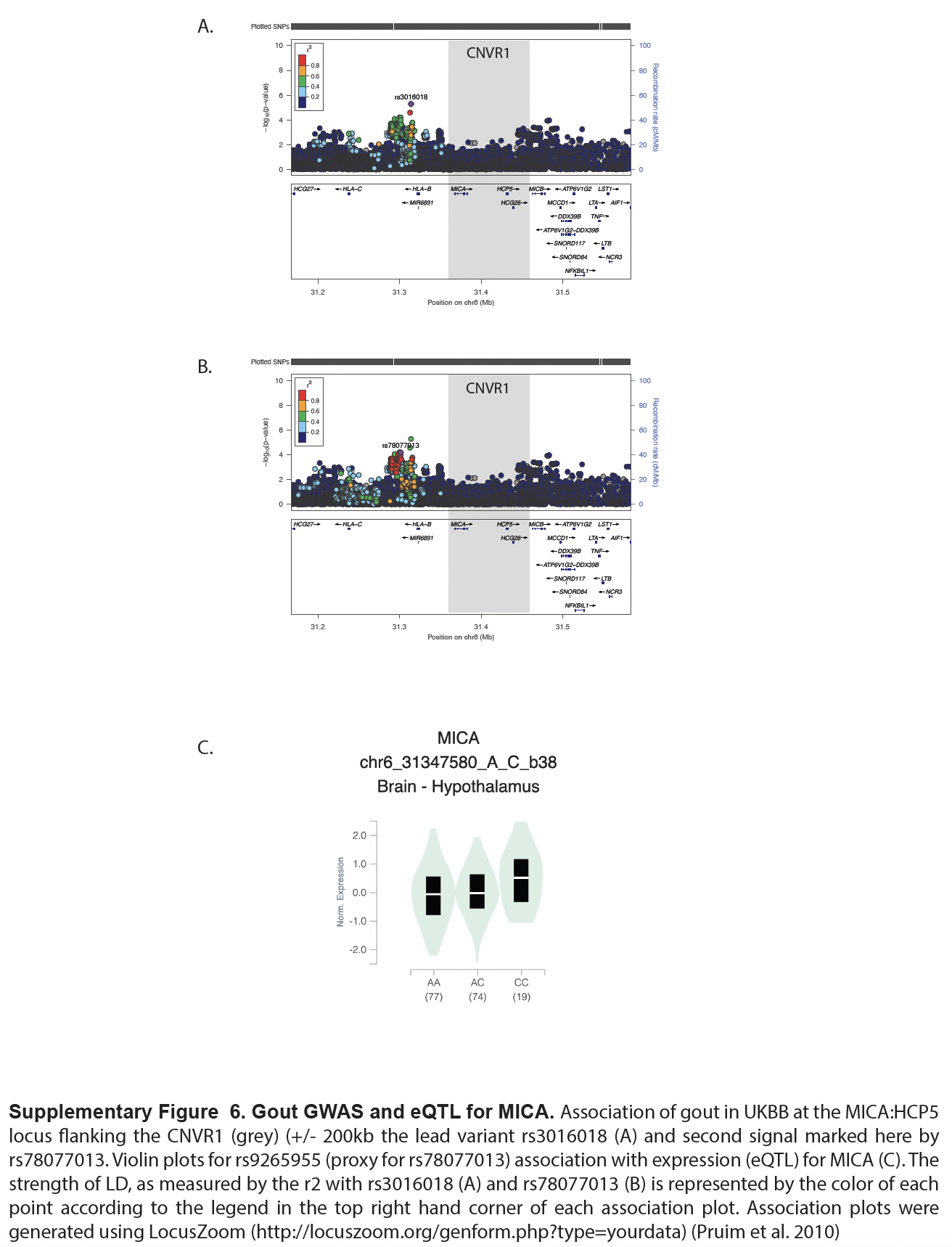


Figure S7. Principal component plots for batch effect on the LRR values of probes.


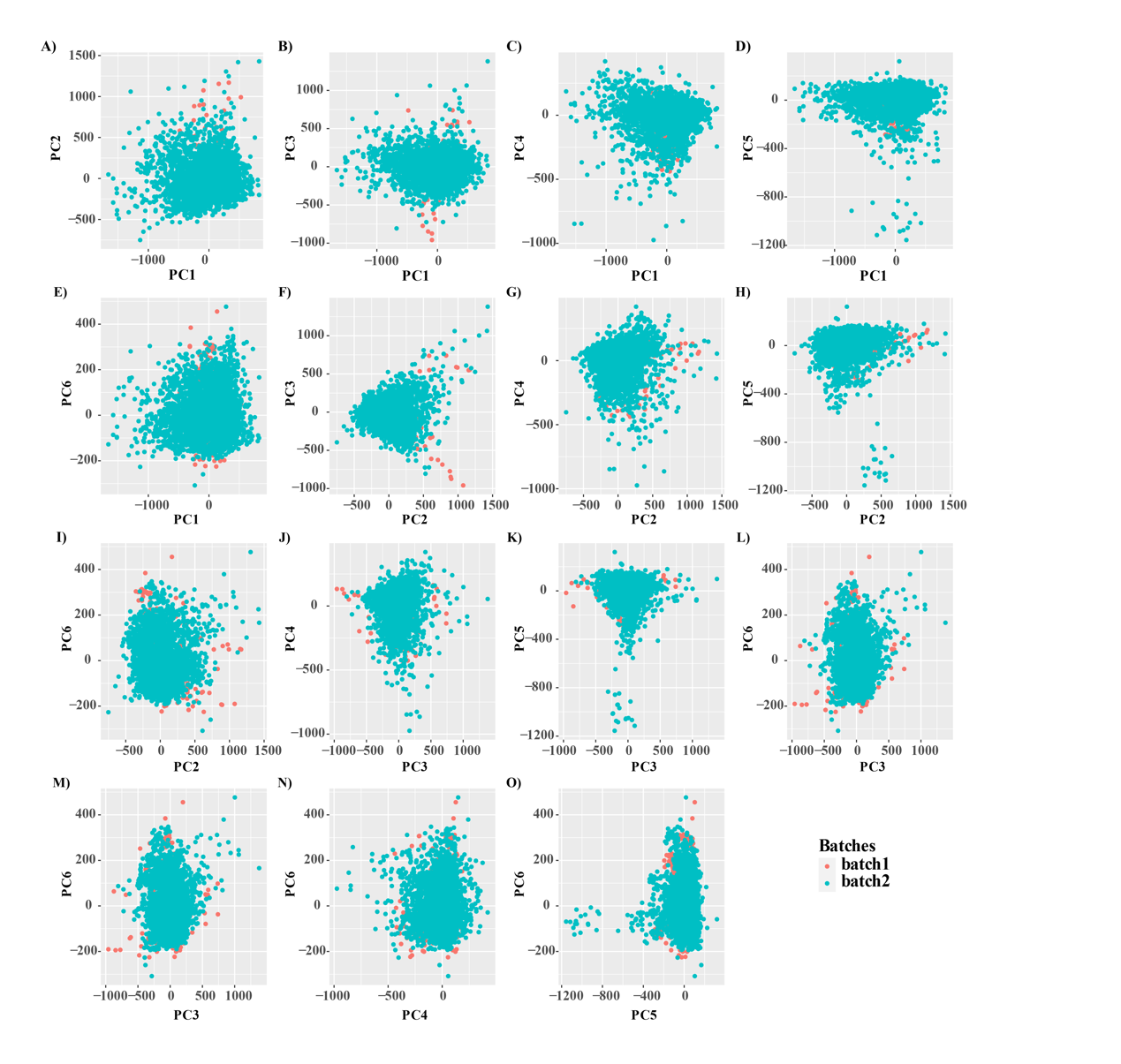


Figure S8. Principal component plots for ethnicity on the LRR values of probes.


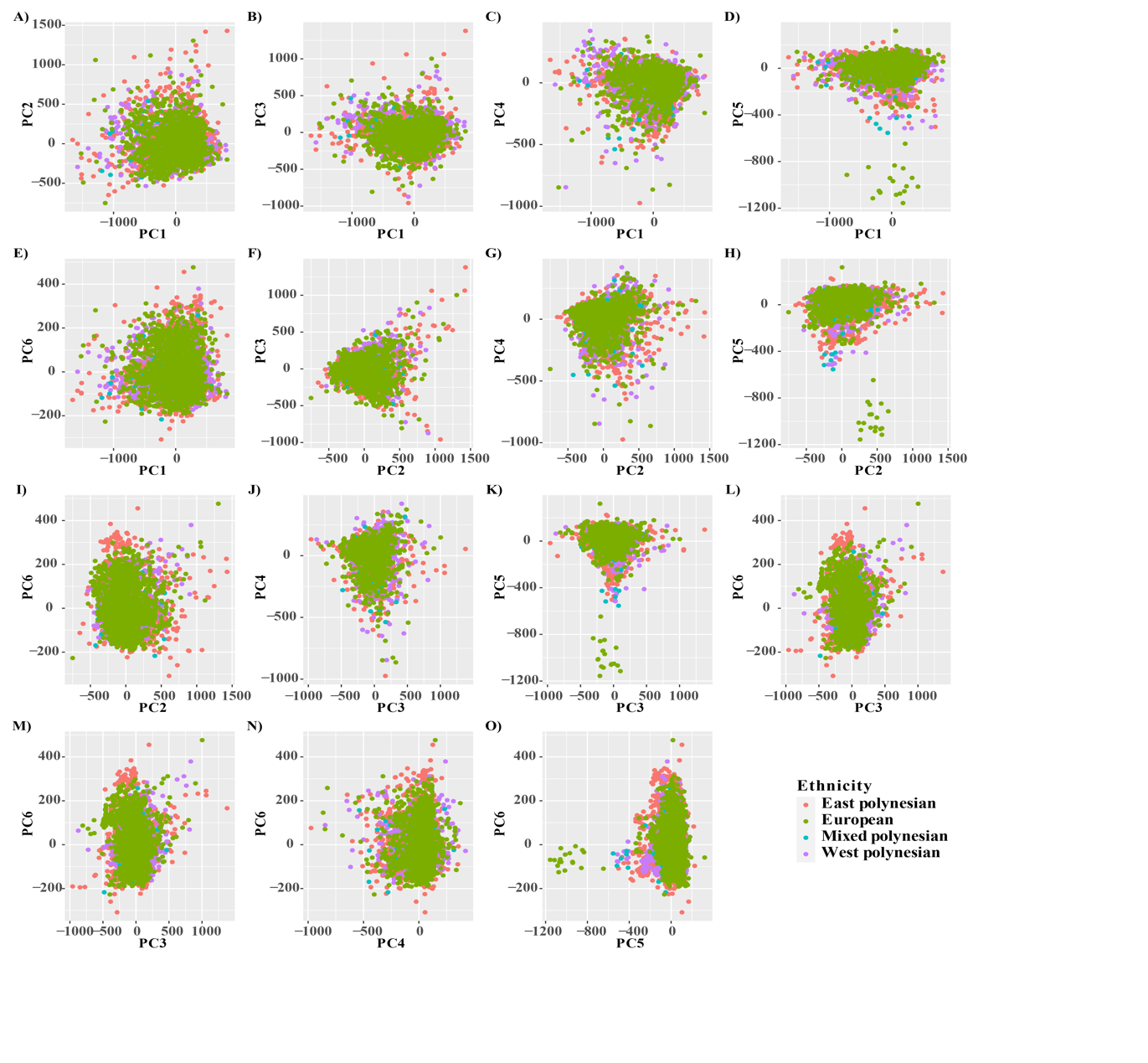


Figure S9. Workflow of CNV detection and filtering, and of how various sample sets were used in the study.

HMM, hidden Markov model; PFB, Population frequencies of the B allele; LRR, Log R Ratios.


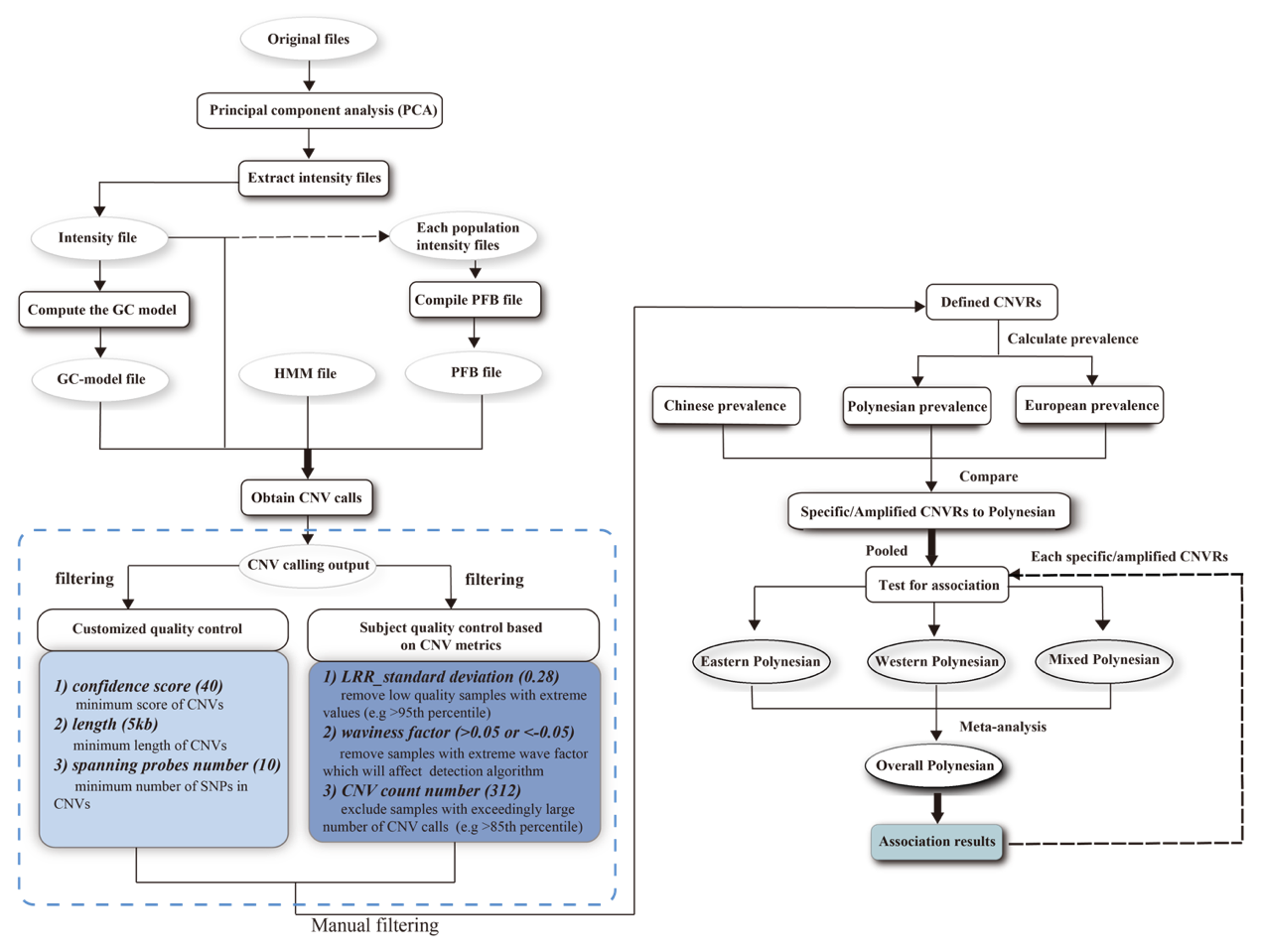


Figure S10. Distribution of number of CNV calls.


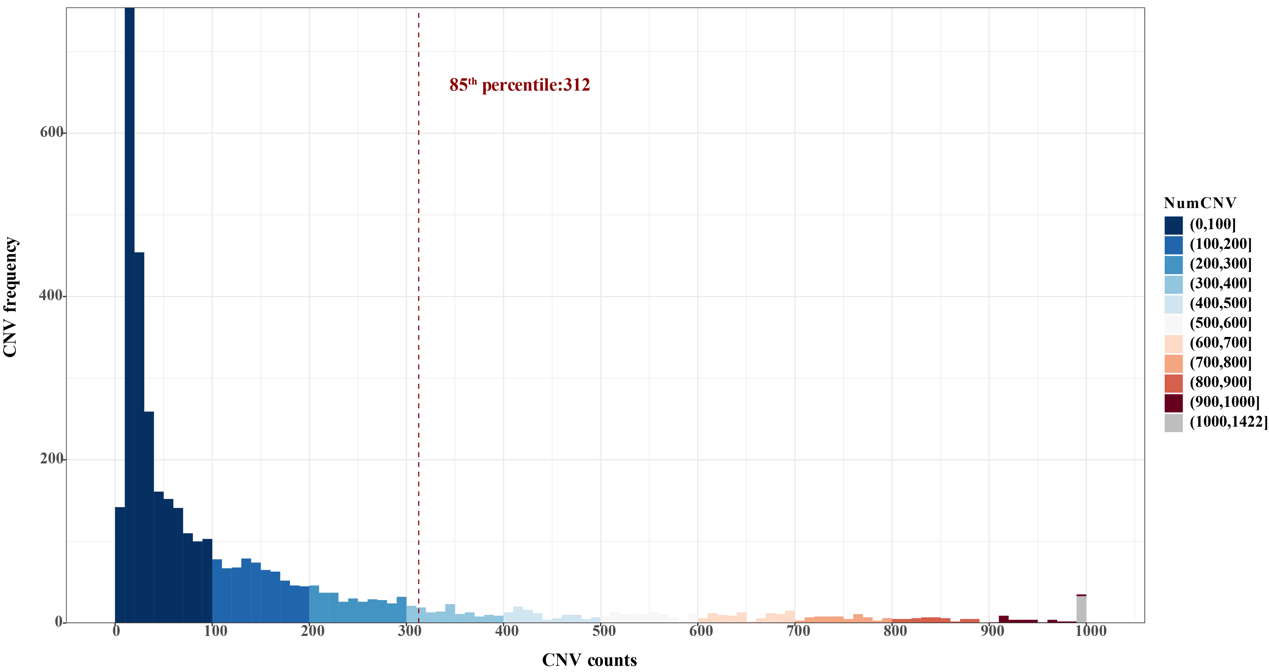

Supplement: PolynesianCNV_supplement_material_coauthors_1208_revised_ddac094 [file polynesiancnv_supplement_material_coauthors_1208_revised_ddac094.zip › PolynesianCNV_supplement_material_coauthors_1208_revised_ddac094.docx]
